# Flexible Survival Extrapolation with Blended Hazards: Accounting for Treatment Effect Waning in Health Technology Assessment

**DOI:** 10.1177/0272989X261452264

**Published:** 2026-06-19

**Authors:** Jingqi Zhu, Matthew Hemstock, Zhaojing Che, Gianluca Baio, Richard Birnie

**Affiliations:** Centre for Biostatistics, University of Manchester, Manchester, UK; Source Health Economics, London, UK; Nuffield Department of Population Health, University of Oxford, Oxford, UK; Department of Statistical Science, University College London, London, UK; Lumanity, Sheffield, UK

**Keywords:** survival extrapolation, treatment effect waning, external evidence, real world evidence, health technology assessment

## Abstract

**Background:**

Survival extrapolation is crucial in estimating the lifetime survival benefit of a treatment for health technology assessment (HTA). Conventional extrapolation methods, which assume that the long-term treatment effect (hazard ratio between treatment and comparator) follows the same pattern as observed in the short-term trial, have been challenged by a wide range of immuno-oncology therapies, particularly those with administrative stopping rules that mandate treatment discontinuation at a prespecified time point. A gradual waning of their treatment effects has been considered plausible and received growing attention from HTA stakeholders over the past decade. However, existing statistical methods often rely on unnecessarily strong waning assumptions.

**Objective:**

We demonstrate the blended hazard method as a flexible way to account for treatment effect waning while incorporating external evidence in survival extrapolation.

**Method:**

The blended hazard method fits separate parametric survival regression models to the observed randomized controlled trial data and external data that inform the common long-term hazard when there is no treatment effect. For each arm, the fitted internal and external hazard functions are blended based on a time-varying weight function, so that the blended hazard is initially dominated by the fitted internal hazard, then gradually approaches the fitted external hazard over a blending interval, and is finally dominated by the fitted external hazard. The time and rate of blending the internal and external information can be controlled by the weight function to allow for sensitivity analyses. NICE TA366 on pembrolizumab for advanced melanoma not previously treated with ipilimumab is used as a case study to demonstrate the practical implementation of this method.

**Results:**

Extrapolations and restricted mean survival times from the blended hazard method closely matched the updated 7-y trial follow-up and showed better consistency than the TA366 base case across all sensitivity analysis scenarios.

**Conclusion:**

The method explicitly accounts for gradual treatment effect waning while incorporating external evidence and offers the flexibility to accommodate a broad range of waning scenarios, thereby effectively characterising uncertainty in extrapolation.

**Highlights:**

- Treatment effect waning is considered plausible in survival extrapolation for many therapies, particularly those with treatment-stopping rules. However, there is a shortage of appropriate methods to model this phenomenon, and existing approaches either rely on strong waning assumptions or address it only as a post hoc check.
- We demonstrate the blended hazard method as a possible approach to account for treatment effect waning while incorporating external evidence.
- The blended hazard method possesses the flexibility to accommodate a wide range of waning scenarios, thereby relaxing unnecessarily strong assumptions and effectively characterizing uncertainty in survival extrapolation.

In many jurisdictions, health care decision making is informed by economic evaluation in the form of cost-effectiveness analysis, which compares the costs and effects of interventions over an appropriate time horizon of affected individuals. For life-extending interventions, this involves adopting a lifetime horizon. A large proportion of these analyses rely on survival data from randomized controlled trials (RCTs), where follow-up is often too short to fully capture lifetime survival outcomes. As a result, survival extrapolation is typically unavoidable in health technology assessment (HTA).^1^ In oncology, partitioned survival models, which estimate the proportion of patients in different health states via independently modeled survival curves, are commonly used because they allow the direct use of aggregate outcomes such as overall survival (OS) and progression-free survival from RCTs.^2,3^ Reliable extrapolation of these survival curves is therefore crucial for health care decision making.

Conventional survival extrapolation methods for partitioned survival models typically assume that long-term treatment effects mirror those observed in the short-term RCT.^4^ However, advanced therapeutic medicinal products, such as chimeric antigen receptor T-cell (CAR-T) therapies and immune checkpoint inhibitors, challenge this assumption due to their complex therapeutic dynamics.^5,6^ One contributing factor is treatment effect waning, whereby relative treatment effects, often expressed as hazard ratios (HRs), are thought to attenuate gradually over several years following treatment discontinuation or disease progression beyond the trial follow-up period.^7^

This challenge is further compounded by the widespread use of administrative stopping rules, which discontinue the treatment at a prespecified time point in many immuno-oncology (IO) regulatory protocols and technology appraisals (TAs).^8^ These rules are motivated by the mechanism of action of IO therapies, which can induce durable responses by activating the patient’s immune system to recognize and attack tumor cells while retaining immune memory for long-term surveillance.^9,10^ Although stopping rules can reduce treatment costs, they may also diminish population-level life-year benefits if applied to patients with incomplete or nondurable responses.^11^ Consequently, failing to account for treatment effect waning under stopping rules risks producing overly optimistic cost-effectiveness estimates and suboptimal resource allocation.

The importance of modeling treatment effect waning has been increasingly recognized over the years. The UK HTA agency, National Institute for Health and Care Excellence (NICE), requires consideration and justification of waning assumptions in the user guide for the company evidence submission template, although the user guide does not specify what method should be implemented.^12^ The NICE Decision Support Unit (DSU) produces Technical Support Document (TSDs) to provide methodological recommendations for HTA submissions. NICE DSU TSD 14 recommends that modelers evaluate at least 3 scenarios: no treatment effect beyond the trial, lifetime maintenance of treatment effect, and treatment effect waning over a finite period.^13^ NICE DSU TSD 21 further emphasizes that extrapolations based on appropriate predefined waning assumptions can be considered plausible.^1^ Similarly, the Canadian HTA agency, Canada’s Drug Agency (CDA-AMC), advises the consideration of the same 3 scenarios as outlined in NICE DSU TSD 14 and highlights that the scenario with treatment effect waning is generally the most plausible.^14^ However, no HTA body, including NICE and CDA-AMC, has yet provided any specific statistical methodology on how the waning mechanism should be modeled.

Reviews of NICE TAs for IO therapies find that waning assumptions have appeared in many company submissions, external assessment group (EAG) reports, and appraisal committee comments.^7,8,11,15^ Existing approaches generally fall into 2 categories. The first assumes proportional hazards and sets the HR abruptly to one from a specified time point onward.^7,8,11^ The second allows the HR to converge linearly or log-linearly to one over a specified time interval and remains at one thereafter.^7,16,17^ Both approaches rely on strong and untestable assumptions: abrupt changes in HR can produce clinically implausible hazard and survival shapes, while linear or log-linear convergence lacks the flexibility to represent uncertainty in waning timing and rate.

In this article, we show how the blended framework, originally proposed by Che et al.^18^ to incorporate external evidence for survival extrapolation, can be extended to model treatment effect waning. Specifically, their framework combines models fitted to RCT and external data via a weight function to achieve plausible extrapolation and sufficient flexibility and can be implemented on both the survival and hazard scales. Here, we make further adjustments to the hazard-scale version to accommodate different assumptions about the rate and time of treatment effect waning and refer to the resulting approach as the blended hazard method. We present its application to account for the potential treatment effect waning described in NICE TA366,^19^ based on OS data available at the time of the initial appraisal. Extrapolations and restricted mean survival times (RMSTs) from the blended hazard method show strong consistency with the updated 7-y trial follow-up and represent a notable improvement over the TA366 base case across all scenarios in the sensitivity analyses. The R code for the full implementation of the method is available at https://github.com/JZhu919/BlendTrtWaning. An interactive Shiny App illustrating the approach is accessible at https://jzhu919.shinyapps.io/shinyapp/.

## Motivating Case Study

NICE TA366^19^ is identified as a motivating case study due to the immaturity of RCT data at the time of appraisal and the plausible presence of treatment effect waning. NICE TA366 evaluated pembrolizumab for advanced melanoma in patients not previously treated with ipilimumab. The main trial was KEYNOTE-006, a phase III trial comparing pembrolizumab (10 mg/kg every 2 wk or every 3 wk up to 2 y) with ipilimumab (3 mg/kg every 3 wk for 4 doses). OS data from the second interim analysis (IA2) were used for the initial appraisal, with a median follow-up of 13.84 mo and a minimum follow-up of 12 mo for all patients. At that time, a substantial proportion of patients were censored; the median OS had not been reached in any treatment arm.^20^ The KEYNOTE-006 protocol prespecified a 2-y stopping rule for pembrolizumab. But it was not enforced due to limited follow-up. As a result, the EAG was cautious about the consequences of the stopping rule on the OS benefit of pembrolizumab, and the stopping rule was not accepted by the appraisal committee due to the limited clinical evidence.

In TA366, base-case OS extrapolations for both treatment arms were presented via a piecewise approach that combined data from the KEYNOTE-006 with 2 external sources. The first was a pooled analysis of treatment-naive melanoma patients treated with ipilimumab with 7 y of follow-up by Schadendorf et al.^21^ The second was registry data from the American Joint Committee on Cancer (AJCC) melanoma staging study by Balch et al.^22^

The base-case extrapolation used the Kaplan–Meier estimate from KEYNOTE-006 for the first 12 mo, a standard parametric model fitted to the Schadendorf dataset from 12 mo to 7 y, and a further model fitted to the AJCC registry data from 7 to 30 y. However, this piecewise approach resulted in the estimated hazard changing erratically at the time points where model pieces join together. Moreover, because both treatment arms were informed by the same external data beyond 12 mo, the implied HR suddenly shifted to one at 12 mo and remained thereafter. Consequently, the EAG and appraisal committee raised concerns related to the clinical plausibility of the resulting hazard patterns.

In this study, we show how the blended hazard method can overcome the limitations of the piecewise approach and capture a gradual treatment effect waning by the 2-y stopping rule for pembrolizumab.

## Methodology

The framework proposed by Che et al.^18^ provides a principled way to combine fitted internal and external curves on either the survival or hazard scale. In this work, we adopt the hazard-scale formulation because it models the arm-specific hazard functions directly and better represents how the relative treatment effect—HR between treatment arms—evolves over time.

The blended hazard method fits separate parametric survival regression models to the observed main RCT data (the internal data) and to external data that inform the long-term hazard when there is no treatment effect (the external data). The modeled hazard functions are called “fitted internal hazard” and “fitted external hazard,” respectively. The blended hazard of each arm begins by following the fitted internal hazard; the time-varying weight function then induces a smooth transition toward the fitted external hazard over the blending interval, after which the external hazard fully governs long-term behavior.

Specifically, let $h_{\mathrm{in}t_{i}}\left(t|\theta_{\mathrm{in}t_{i}}\right)$ denote the fitted internal hazard for arm *i* ( i=1 for treatment and i=0 for comparator), and let $h_{\mathrm{ext}}\left(t|\theta_{\mathrm{ext}}\right)$ denote the fitted external hazard. Then, the blended hazard function for arm i is defined as a time-varying weighted average of the 2 fitted hazard functions:

$$\begin{array}{c} h_{\mathrm{blen}d_{i}}\left(t|\theta_{i}\right)=\left[1-\pi \left(t|t_{1},t_{2},a,b\right)\right]\times h_{\mathrm{in}t_{i}}\left(t|\theta_{\mathrm{in}t_{i}}\right)\\ +\pi \left(t|t_{1},t_{2},a,b\right)\times h_{\mathrm{ext}}\left(t|\theta_{\mathrm{ext}}\right) \end{array}$$

where $\theta_{i}=\left(\theta_{\mathrm{in}t_{i}},\theta_{\mathrm{ext}},t_{1},t_{2},a,b\right)$ .

The weight function $\pi \left(t|t_{1},t_{2},a,b\right)$ governs how the rate of blending changes over the blending interval $\left(t_{1},t_{2}\right)$ and is defined as the cumulative distribution function of a Beta(a,b) distribution evaluated at $\left(t-t_{1}\right)/\left(t_{2}-t_{1}\right)$

$$\pi \left(t|t_{1},t_{2},a,b\right)=\left\{ \begin{array}{c} 0\;\mathrm{for}\;0\leq t<t_{1}\\ F_{\mathrm{Beta}}\left(\frac{t-t_{1}}{t_{2}-t_{1}}\;\;a,b\right)\;\mathrm{for}\;t_{1}\leq t<t_{2}\\ 1\;\mathrm{for}\;t\geq t_{2} \end{array}\right.$$

where $F_{\mathrm{Beta}}$ is the Beta cumulative distribution function.

The relative treatment effect at time t is given by

$$\mathrm{HR}\left(t|\theta_{0},\theta_{1}\right)=\frac{h_{\mathrm{blen}d_{1}}\left(t|\theta_{1}\right)}{h_{\mathrm{blen}d_{0}}\left(t|\theta_{0}\right)}$$

**Figure 1:**
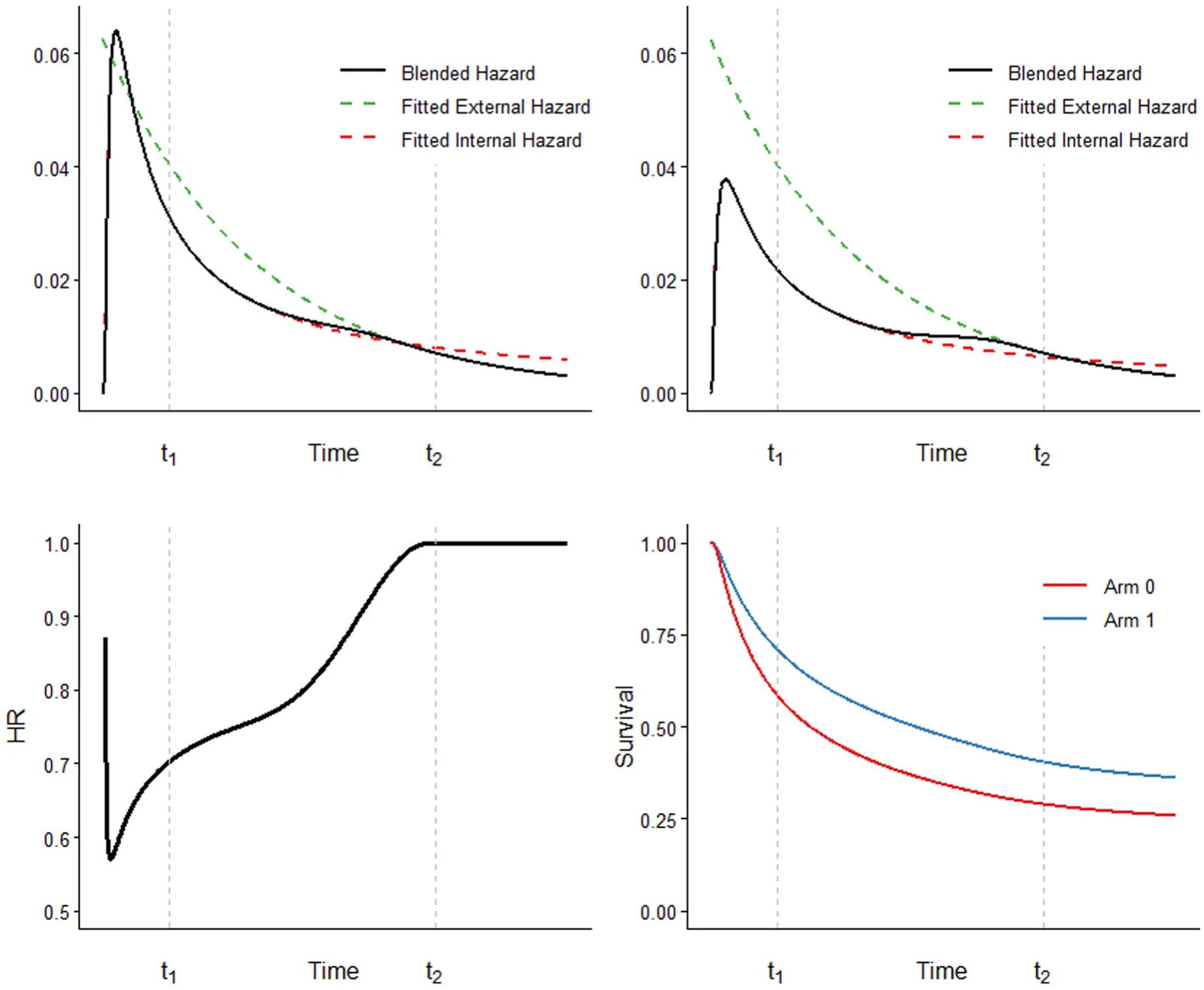
Illustration of the blended hazard method. (A–B) Hazards for each arm (A for arm 0, B for arm 1) start by following the fitted internal hazard and progressively transition to the external hazard over the blending interval. (C) The hazard ratio (HR) increases toward 1 as the hazards converge during the blending interval and remains at 1 afterward. (D) Survival curves obtained from the blended hazards are smooth and run parallel in the long term.

Figure 1 illustrates the blended hazard method. For each treatment arm, the hazard follows the internal model at early times and the external model at long times, with a smooth transition induced by the weight function over the blending interval (panel A for arm 0, panel B for arm 1). During this interval, the HR is gradually attenuated toward 1 and remains at 1 thereafter (panel C). The method is flexible and can accommodate irregular or nonstandard patterns of treatment effect waning. The trajectory of the HR is partly data driven: it depends not only on the shapes of the fitted internal and external models but also on how they are blended together. This construction ensures continuity of the hazard functions and produces smooth survival curves that eventually run in parallel (panel D), which may be considered more clinically plausible than models that impose abrupt changes in hazard.

Identifying suitable external data is crucial. Ideally, the external source should involve the same comparator, indication, and target population and include sufficiently long follow-up to inform the hazard at time points where the treatment and comparator are expected to converge. In other words, the external dataset should represent the long-term hazard that is ultimately common to all treatment arms.

Careful clinical and statistical consideration is required when specifying the 4 key components of the blended hazard framework: the fitted internal model $h_{\mathrm{in}t_{i}}\left(t|\theta_{\mathrm{in}t_{i}}\right)$ , the fitted external model $h_{\mathrm{ext}}\left(t|\theta_{\mathrm{ext}}\right)$ , the blending interval $\left(t_{1},t_{2}\right)$ , and the Beta parameters (a,b) .

Conventional model selection for trial-based extrapolation has to balance short-term fit with plausible long-term projections. Under the blended hazard framework, however, the internal model can prioritize accurately capturing the short-term RCT data, since the external model is explicitly responsible for informing the long-term hazard behavior. Accordingly, the external model should be chosen to best represent the long-term hazard patterns. Its fit to very early observations that overlap with the trial period is less consequential, as the external hazard receives zero weight during that time. The weight function performs most of the heavy lifting by regulating the gradual transfer of influence from the internal to the external hazard, thereby controlling the shape of blended hazard, survival, and HR.

The start of the blending interval $t_{1}$ marks the time beyond which the fitted internal hazard is no longer regarded as fully reliable. The end of the interval $t_{2}$ represents the point at which the 2 arms are assumed to have equal hazards. These time points are typically informed by the maturity of the observed RCT data, clinical expertise, or a focused review of historical treatments within the same therapeutic area.

The Beta parameters a and b define the shape of the weight function over the blending interval and therefore determine the rate of the blending fitted internal and external information. This should be distinguished from the rate of treatment effect waning, which is reflected in the evolution of the HR between the arms. Given the substantial uncertainty around waning assumptions, multiple scenarios for both the blending interval and the Beta parameters should be examined in sensitivity analyses.

## Implementation

In our TA366 case study, the internal data comprised OS data for both the pembrolizumab and ipilimumab arms from the KEYNOTE-006 interim analysis used in the original appraisal.^20^ We used the OS data for treatment-naive patients from the Schadendorf pooled analysis^21^ as the external data, which provide 7 y of follow-up and were also used as external data in the TA366 base-case extrapolation. Because the relative treatment effect of pembrolizumab versus ipilimumab is expected to have diminished by 7 y, these external data were deemed informative about the eventual long-term common hazard in both arms. Individual patient data for both internal and external datasets were reconstructed by digitizing the published Kaplan–Meier estimates and applying the method by Guyot et al.^23^

### Internal Model Selection

For each treatment arm, we first experimented with standard parametric models recommended in NICE DSU TSD 14^13^ and then progressively increased model complexity by incorporating 1-knot Royston–Parmar splines (hazard, odds, and normal models),^24^ followed by 2-knot splines, and ultimately 3-knot splines, until an adequate fit was achieved. Model selection was based on internal validation, including visual comparison of fitted hazards with smoothed hazard estimates, visual comparison of fitted survival curves with the Kaplan–Meier functions, and the Akaike information criterion (AIC) and Bayesian information criterion (BIC). Because smoothed hazard estimates become increasingly uncertain near the end of follow-up due to small numbers at risk, we placed greater attention on goodness of fit before the median follow-up of the internal data (13.84 mo) when visually evaluating the hazard functions.

### External Model Selection

We explored standard parametric models from randomization and models rebased at 13.84 mo, which corresponds to the median follow-up of the internal data. Rebasing was considered for 2 reasons: first, the Kaplan–Meier of the external data was supported by a substantial number of events beyond this time, providing a reliable basis for modeling the subsequent hazard; second, we believed that the fitted internal hazard should be sufficiently reliable before this time such that there is no need to blend in external information. Therefore, the rebased models can focus on characterizing mid- and long-term hazard beyond this point. All candidate models were compared based on visual inspection of hazard and survival fits, as well as AIC and BIC.

### Blending Interval

Two scenarios were considered for the start of the blending interval $t_{1}$ : 14 mo (immediately following the median internal follow-up of 13.84 mo) and 24 mo (corresponding to administrative treatment discontinuation under the previously proposed 2-y stopping rule). For the end of the blending interval $t_{2}$ , we drew on 2 review articles by Kamgar et al.^8^ and Taylor et al.,^11^ which summarized NICE IO appraisals incorporating both treatment effect waning assumption and stopping rules. These reviews suggest that a 3- to 5-y treatment effect waning period after treatment initiation is generally regarded plausible by the appraisal committee. Based on this pattern, 36 mo and 60 mo were selected as 2 scenarios for the end of the blending interval $t_{2}$ . Combining choices for $t_{1}$ and $t_{2}$ yielded 4 scenarios for the blending interval $\left(t_{1},t_{2}\right)$ : (14,36) , (14,60) , (24,36) , and (24,60) months.

### Beta Parameters

To reflect uncertainty in the shape of the weight function, we evaluated 4 sets of Beta parameters (a,b) representing distinct blending profiles: 1) (0.2,0.2) for equally small a and b , producing a sharp transition of weights near the ends of the interval; 2) (5,5) for equally large a and b , yielding gradual blending centred within the interval; 3) (3,7) for a>b , resulting in slower blending near the start; and 4) (7,3) for a<b , resulting in slower blending near the end. Combining the 4 blending-interval scenarios with the 4 Beta-parameter scenarios resulted in 16 candidate weight functions $\pi \left(t|t_{1},t_{2},a,b\right)$ , thus 16 models for sensitivity analysis.

Survival extrapolations from all 16 models were compared with the updated 7-y Kaplan–Meier estimates from KEYNOTE-006^25^ and with the TA366 base-case survival extrapolation reproduced using the methodology described in the committee papers.^19^ The 7-y RMST was calculated for each model by integrating under the updated Kaplan–Meier curve or the corresponding extrapolated curve. The model representing the most clinically plausible hazard and survival is selected as the base case.

## Results

### Selected Models

The selected internal models comprised a 3-knot spline normal model for the pembrolizumab arm and a generalized gamma model for the ipilimumab arm. The selected external model was a Gompertz model rebased at 13.84 mo. The base case was selected to be the model with a blending interval of (24,60) months and Beta parameters (5,5) , that is, weight function $\pi \left(t|t_{1}=24,t_{2}=60,a=5,b=5\right)$ . Full details of the model and base-case selection processes are provided in Supplementary Material S1 and S2.

### Base-Case Analysis

**Figure 2:**
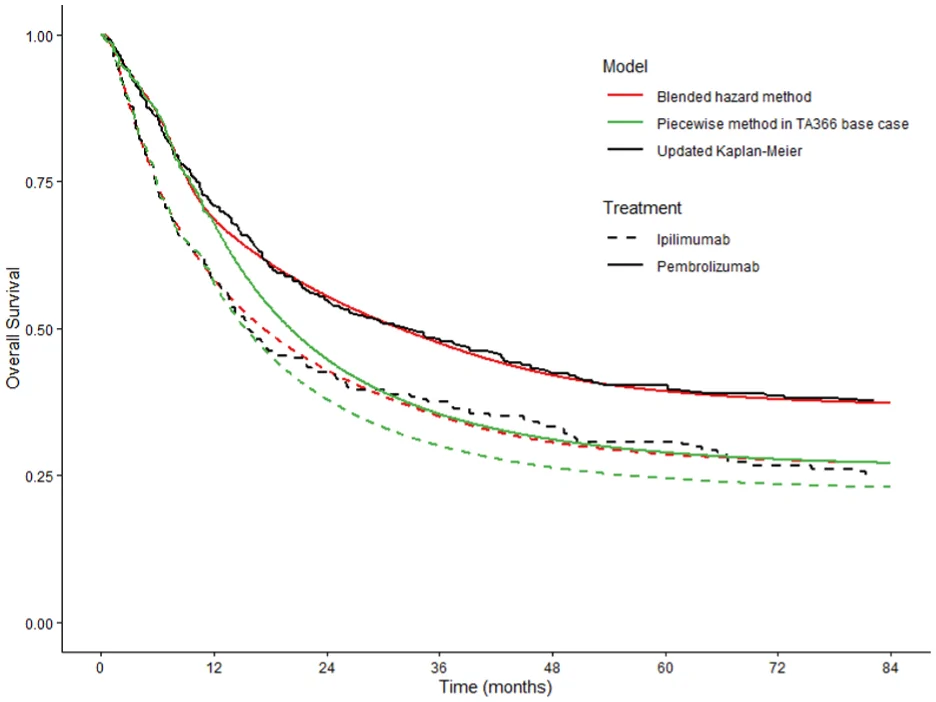
Comparison of survival extrapolations from the blended hazard method base case with the updated 7-y Kaplan–Meier and the piecewise-model extrapolations in the TA366 base case.

Figure 2 compares survival extrapolations from the blended hazard method base case with both the updated 7-y Kaplan–Meier estimates of KEYNOTE-006 and the TA366 base-case piecewise-model extrapolations. The blended hazard method closely reproduced the Kaplan–Meier estimates for both treatment arms and yielded accurate 7-y RMST estimates (Table 1). In contrast, the piecewise model used in the TA366 base case consistently underestimated survival and 7-y RMST in both arms relative to the updated trial data, with the discrepancy most pronounced for pembrolizumab. The incremental 7-y RMST of pembrolizumab versus ipilimumab was also underestimated by the piecewise model. The closer agreement of the blended method with the updated 7-y follow-up data suggests that it provides projections that are more consistent with the observed long-term survival patterns than the piecewise model used in the TA366 base case.

**Table 1:** Comparison of 7-Year Restricted Mean Survival Time Estimates from the Blended Hazard Method Base Case, Updated Kaplan–Meier, and the Piecewise Method in the TA366 Base Case

Model	Pembrolizumab	Ipilimumab	Increment
Blended hazard method base case	3.59	2.83	0.76
Updated 7-y Kaplan–Meier	3.61	2.84	0.77
Piecewise method in TA366 base case	2.98	2.57	0.41

### Sensitivity Analyses

Figures that compare survival extrapolation between the blended hazard method, the updated 7-y Kaplan–Meier, and the piecewise-model extrapolations in the TA366 base case for the 15 sensitivity analysis scenarios are accessible at https://github.com/JZhu919/BlendTrtWaning/tree/main/figures/sensitivity%20analysis. Table 2 summarizes the 7-y RMST estimates across all 16 scenarios, contrasting them with estimates derived from the updated 7-y Kaplan–Meier and the piecewise extrapolation in the TA366 base case.

**Table 2:** Seven-Year Restricted Mean Survival Time Estimates across All 16 Scenarios of the Blended Hazard Method, Compared with Estimates Derived from the Updated 7-Year Kaplan–Meier and the TA366 Base-Case Piecewise Extrapolation

$\left(t_{1},t_{2}\right)$	(a,b)	Pembrolizumab	Ipilimumab	Increment
(14, 36)	(0.2, 0.2)	3.25	2.69	0.56
(14, 36)	(5, 5)	3.43	2.78	0.65
(14, 36)	(7, 3)	3.52	2.82	0.70
(14, 36)	(3, 7)	3.31	2.73	0.58
(14, 60)	(0.2, 0.2)	3.37	2.73	0.64
(14, 60)	(5, 5)	3.56	2.83	0.74
(14, 60)	(7, 3)	3.58	2.82	0.77
(14, 60)	(3, 7)	3.46	2.79	0.67
(24, 36)	(0.2, 0.2)	3.51	2.81	0.70
(24, 36)	(5, 5)	3.53	2.82	0.71
(24, 36)	(7, 3)	3.56	2.83	0.73
(24, 36)	(3, 7)	3.50	2.81	0.69
(24, 60)	(0.2, 0.2)	3.52	2.79	0.73
(24, 60)	(5, 5)	3.59	2.83	0.76
(24, 60)	(7, 3)	3.58	2.81	0.77
(24, 60)	(3, 7)	3.56	2.83	0.73
Updated 7-y Kaplan–Meier		3.61	2.84	0.77
Piecewise method		2.98	2.57	0.41

Survival extrapolations generated using the blended method consistently followed the updated 7-y Kaplan–Meier estimates for both treatment arms across all scenarios. However, they tended to slightly underestimate the 7-y RMSTs and incremental RMST. This underestimation was typically greater for the pembrolizumab arm than for the ipilimumab arm, and it appeared more pronounced when $t_{2}$ were small (the blending interval commenced and terminated earlier, e.g., $\left(t_{1},t_{2}\right)=\left(14,36\right)$ ) or when the Beta parameters were small (sharp transition of weight function near both ends of the blending interval, e.g., (a,b)=(0.2,0.2) ). All scenarios demonstrated closer adherence to the updated 7-y Kaplan–Meier and yielded more accurate RMST estimates compared with the piecewise extrapolation in the TA366 base case.

## Discussion

### Interpretation of Results

**Figure 3:**
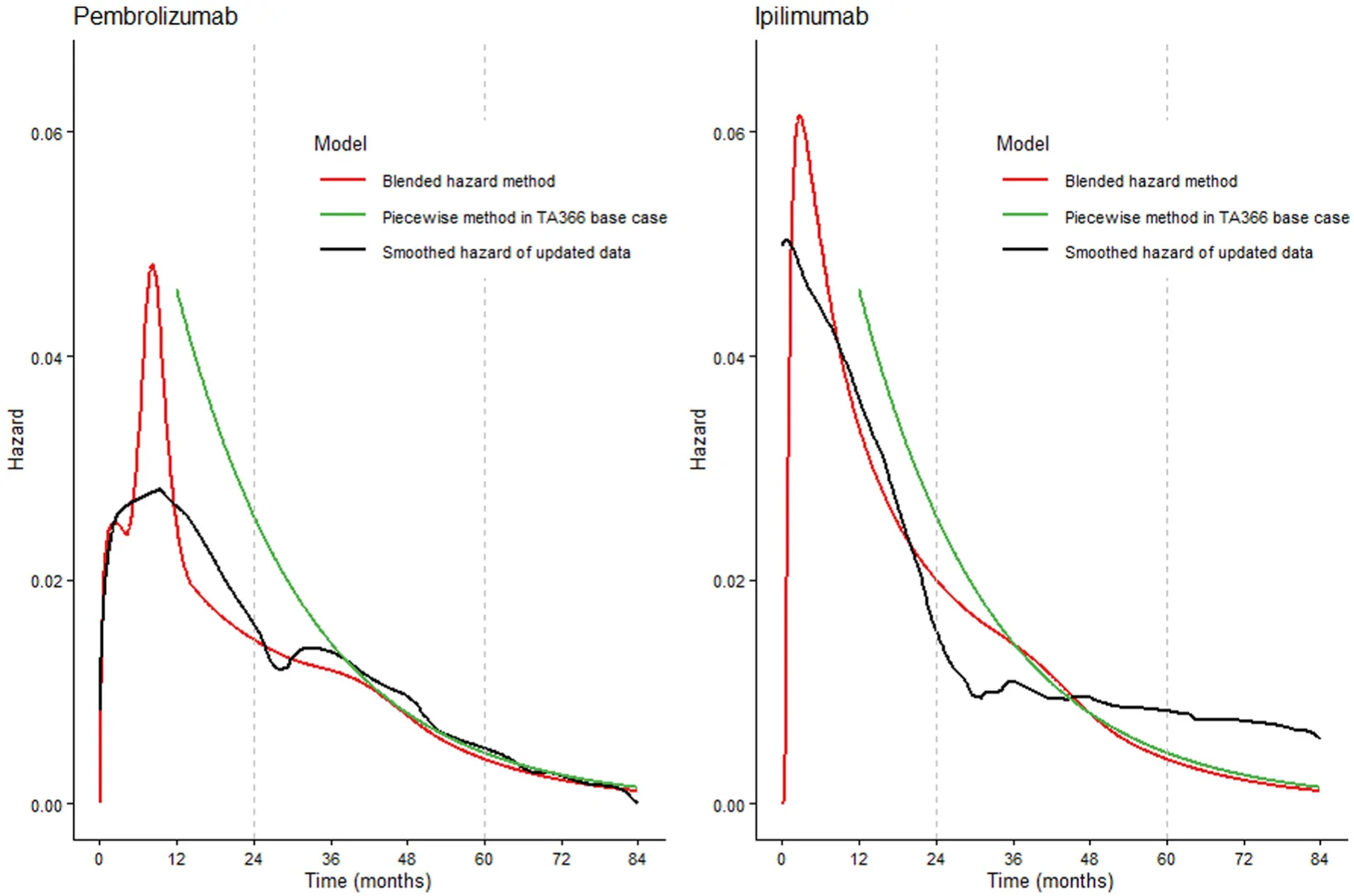
Comparison between the hazard function of the blended method base case, the nonparametric smoothed hazard of the updated 7-y data (pseudo individual patient data estimated from the Kaplan–Meier curve), and the piecewise hazard estimates of the TA366 base case.

The robust extrapolation performance of the blended hazard method is better elucidated when viewed on the hazard scale. Figure 3 illustrates a comparison between the hazard function of the blended method base case, the nonparametric smoothed hazard of the updated 7-y data (pseudo individual patient data estimated from the Kaplan–Meier curve via the Guyot algorithm^23^), and the piecewise hazard estimates of the TA366 base case. The blended hazard tracks closely to the long-term smoothed hazards derived from the updated data for both treatment arms. While some fluctuating differences are observed for the ipilimumab arm, as the hazard is low during that time, these have a negligible impact on the OS curve.

**Figure 4:**
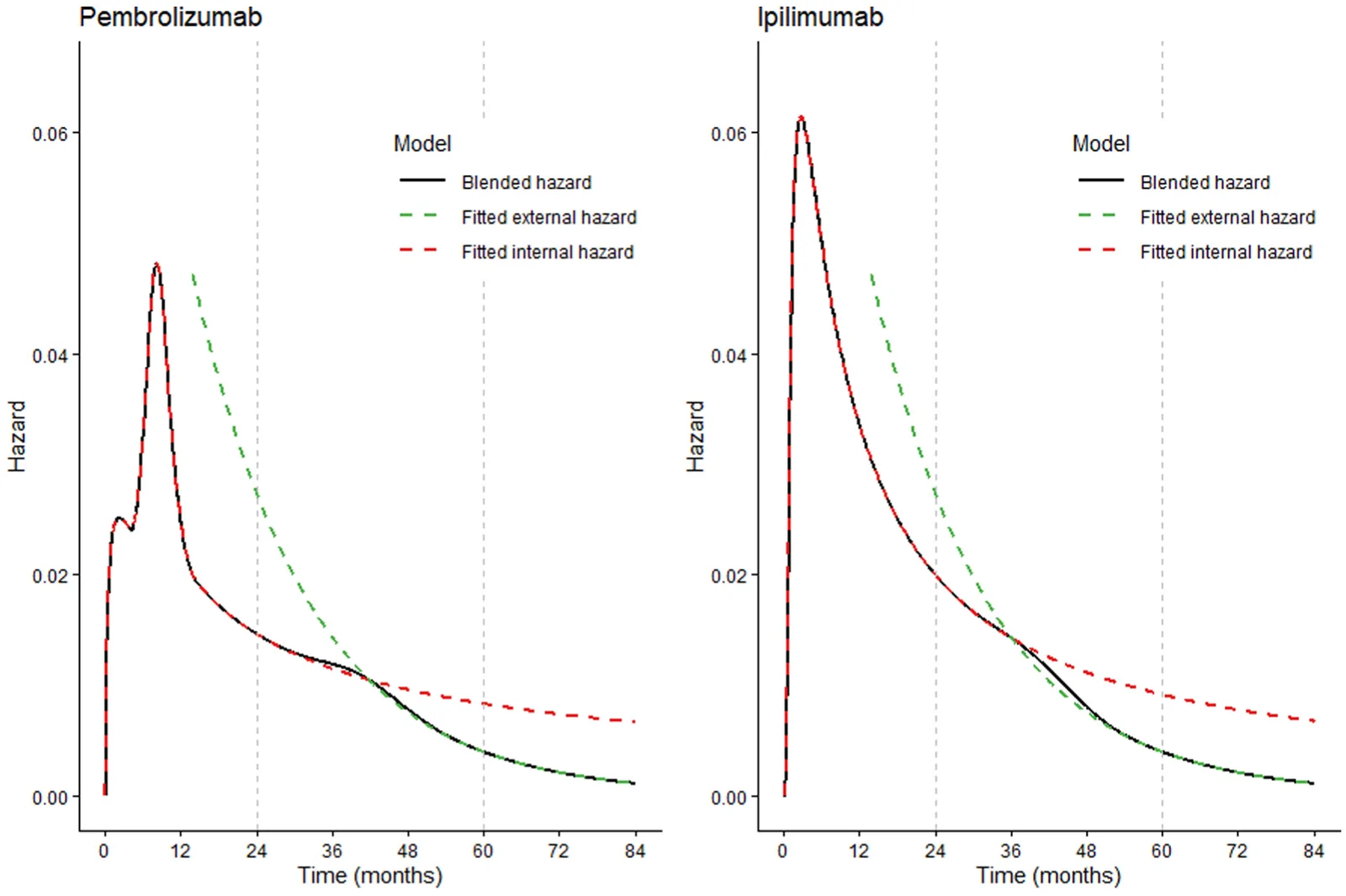
Comparison between the hazard function from the blended method base case, fitted internal model, and fitted external model.

The gradual transition from internal to external evidence of the blended hazard method provides plausible long-term hazard and survival estimates. As shown in Figure 4, the fitted internal hazard is gradually steered downward to match mortality observed in long-term external data. Without this blending process, the trial-based extrapolation (fitted internal model) would overestimate the long-term hazard, thereby leading to underestimated survival for both treatment arms.

In addition, the blended hazard method provides a plausible change of HR. As the blended hazard functions for different treatment arms converge to the same external hazard function, the HR smoothly increases to 1, independent of how each individual treatment arm is modeled. The time point at which the HR reaches 1 can be controlled to reflect specific assumptions regarding treatment effect waning.

In contrast, the piecewise hazard estimate relies exclusively on external data after 12 mo, which overestimated the hazard for 2 y beyond the observed trial follow-up for both treatment arms. This results in systematically underestimated survival curves, particularly for the pembrolizumab arm. Consequently, the incremental OS benefit of pembrolizumab over ipilimumab is underestimated by approximately 47% over the 0- to 7-y time horizon.

Furthermore, as the piecewise model lacks any transition between the 0- to 12-mo internal Kaplan–Meier estimate and the post–12-mo model fitted to the external data, it implicitly assumes a sudden change in the HR to 1 at the 12-mo mark, which is clinically implausible. In fact, given the retrospective nature of this review, the implicit treatment effect waning assumption that no further treatment effect persists beyond 12 mo may have been too conservative.

Sensitivity scenarios with a blending interval of (14,60) or (24,60) months yield extrapolations that demonstrate closer alignment with the updated 7-y trial follow-up, suggesting that a prolonged waning period extending up to 5 y is generally favorable. Scenarios with Beta parameters of (7,3) also tend to produce well-aligned extrapolations, whereas some scenarios with Beta parameters of (0.2,0.2) exhibit sharp fluctuations in the hazard function around the 2 ends of the blending interval, which may lack clinical plausibility.

### Strengths

Survival extrapolation with potential treatment effect waning is essentially about forecasting long-term outcomes driven by an underlying biological mechanism that is not fully understood. Differences in waning methodology can lead to substantial variation in projected life-year outcomes.^26^ It is always essential to incorporate all available evidence and appropriately reflect uncertainty.^27^ Differences in waning methodology may lead to considerable variability in estimated life-year outcomes. Current methods are either constrained by strong and mathematically convenient assumptions (proportional hazards, sudden changes or [log-]linear changes in HR), resulting in models that fail to fully capture uncertainty, or they do not incorporate waning assumptions directly into the model, instead addressing them only as a post hoc check.

We demonstrate the blended hazard method as a possible way to relax unnecessarily strong assumptions, integrating both external evidence and treatment effect waning assumptions. As a result, it possesses the ability to accommodate a wider range of waning scenarios than existing methods for treatment effect waning. Compared with conventional approaches that independently model survival extrapolations with external evidence, the blended hazard method can, to some extent, be viewed as a calibration of long-term survival projections based on treatment effect waning assumptions. The method is not computationally intensive and allows flexible parametric survival models to be easily integrated, making it presumably practical and acceptable to practitioners.

Within the blended framework, scenarios varying the blending interval represent uncertainty in blending time, while scenarios varying Beta parameters represent uncertainty in blending rate. Uncertainty in blending time and rate, combined with the inherent uncertainty in fitted internal and external models, constitutes the overall uncertainty in extrapolation. As knowledge of any of these components improves, the extrapolation uncertainty can be updated correspondingly.

The blended framework, as a flexible approach for combining internal and external information, is not restricted to modeling OS directly. It could also be incorporated within a relative survival framework by modeling excess mortality separately for the internal and external data sources and blending the excess hazard functions while deriving background mortality from general population life tables. Such an extension may be particularly relevant in contexts in which long-term survival is expected to converge toward general population mortality, for example in potentially curative settings such as CAR-T cell therapies.

### Limitations

**Figure 5:**
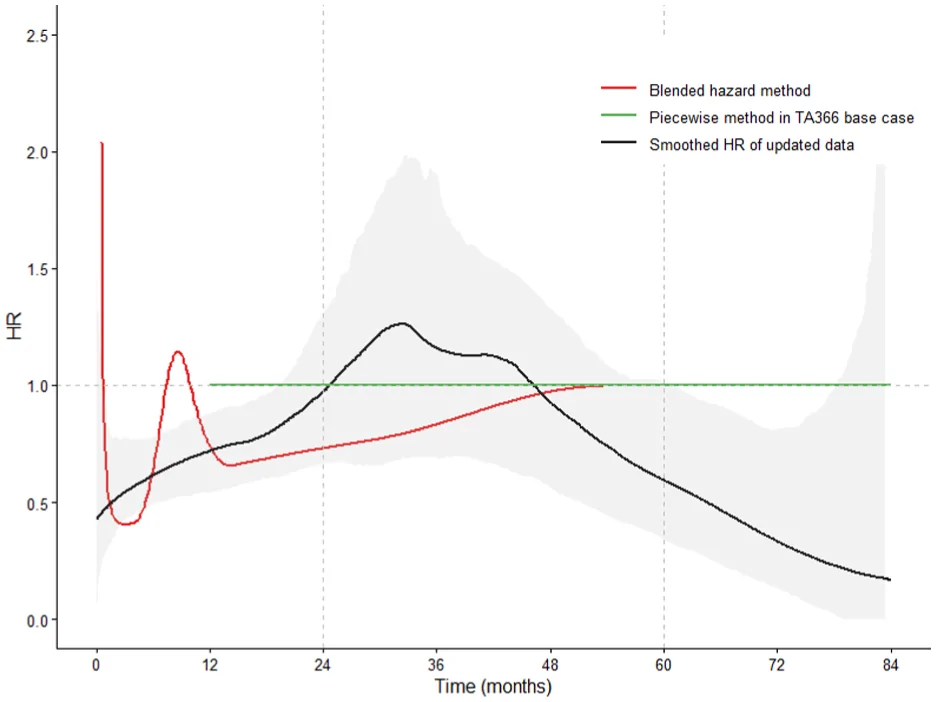
Hazard ratio for pembrolizumab against ipilimumab estimated using the blended hazard method base case (calculated as the ratio of 2 estimated blended hazard functions), the TA366 piecewise method base case, and the smoothed hazard ratio derived from the updated 7-y follow-up data (with 95% bootstrap confidence interval).

Like all other methods for treatment effect waning, the blended hazard method assumes that hazards of different treatments will equalize (HR will become 1) after a certain time point. However, this may not precisely hold in the real world. For example, in our demonstrative case study, the smoothed HR from the updated data is increasing then decreasing, and the bootstrapped 95% confidence interval does not always include 1 after 60 mo (Figure 5). In fact, similar patterns of the HR have been observed in multiple IO NICE TAs, including TA428, TA578, and TA692.^8^ The real-world dynamics of the HR are complex. It can be affected by both the mechanism of action of each treatment^28^ and the subsequent treatments received after the allocated study treatment.^29^ The change in HR may also be attributed to a frailty selection effect^30^: early survival benefits can result in a greater proportion of frailer individuals remaining in the treatment arm at later time points, whereas frailer individuals in the comparator arm may have already died. This implies that constraining the population-level (conditional) HR to 1 may not faithfully represent the absense of individual-level (marginal) treatment effects.^31^ Nevertheless, we believe assuming eventually equalized hazard functions remains a useful and practical modeling assumption, given that all treatment arms are expected to ultimately converge to the background population mortality, and the HR remains the most widely accepted statistical proxy for treatment effect on survival outcomes.

The effectiveness of the blended hazard method has been retrospectively validated on TA366 in this article. To reliably evaluate its long-term utility and robustness, the method should ideally be applied prospectively and subsequently reevaluated for TAs across various treatments and indications.

While the blended hazard method illustrated here integrates internal data with a single source of external data, it may be necessary in practice to incorporate multiple sources of external data to achieve a more robust long-term extrapolation. Attempting to blend multiple sources of external data would constitute a useful area for further research.

A key limitation of the blended hazard method is that modelers need to identify appropriate external data, select the internal and external models, as well as explore a range of blending intervals and Beta parameters, which involves 4 subjective parameters $\left(t_{1},t_{2},a,b\right)$ . The successful implementation of the blended hazard method requires careful consideration toward each component. Below, we provide a concise guidance for reference.

### Guidance for Practical Implementation

#### External data identification

The external data are expected to inform the eventual hazard of both treatment arms when there is no treatment effect. When a novel treatment is compared with an older one with a similar mechanism of action in the same indication, a study of the older treatment with longer follow-up may provide a suitable external data source. Alternatively, studies evaluating the same therapy in the same indication but in later lines of treatment (e.g., second- or third-line use after prior therapy) may also serve as external sources. When none of these options are available, real-world evidence, such as Systemic Anti-Cancer Therapy (SACT)^32^ and National Cancer Registration Dataset (NCRD)^33^ could be used as external data, as they are typically much more mature and better at informing mortality for the general population or some specific subgroups. The framework can also accommodate landmark estimates elicited from clinical experts, which may provide additional information on long-term survival when empirical external data are limited.

#### Population adjustment

The blended hazard method itself does not address potential differences between the internal/external study populations and real-world patients. Oncology RCTs frequently enroll selected patients with more favorable prognostic profiles than those treated in routine practice. Identified external data also often show a divergence in patient characteristics. Where such differences are substantial, additional approaches (e.g., propensity score matching^34^ and g-computation^35^) may be required to improve the generalizability of extrapolated survival estimates.

#### Internal and external model

For both internal and external model selection, we recommend starting with standard parametric models and gradually increasing model complexity until no substantial improvement in goodness of fit is observed—based on visual inspection of hazard and survival plots, as well as AIC and BIC—to avoid unnecessarily complicated models. Royston–Parmar restricted cubic spline^24^ and M-spline^17^ could be useful and practical options to escalate model flexibility.

For the internal model, as explained in Che et al.,^18^ modest overfitting is unlikely to greatly influence long-term projections within the blended framework, because the hazards are constrained to converge toward the fitted external model. In some cases, multiple internal models fit the data almost equally well, and a visual comparison of their long-term extrapolations can help assess their plausibility and guide model selection; choosing an internal model that already implies plausible long-term trends can help reduce sensitivity to the weight function.

For the external model, apart from the standard parametric models, rebased models could be alternative options to concentrate model fit to mid-term and long-term observations beyond the follow-up of internal data. In our study, we set the rebased time point to be the median follow-up of the internal data as an illustrative example. In practice, to employ rebased models, care must be taken to ensure that there are sufficient events beyond the chosen time point to adequately support the external model; otherwise, the reliability of long-term extrapolation may be compromised.

#### Blending interval and Beta parameters

The start of the blending interval depends on the modeler’s belief of when the fitted internal hazard is no longer completely reliable and should be supplemented by external information. When modelers are not very confident in the internal model toward the end of internal trial, the minimum or median follow-up of the internal trial could be used, provided that they are not too short and occur before the Kaplan–Meier tail becomes dominated by study-end censoring. The time of treatment discontinuation can also be considered if the administrative stopping rule has not yet been enforced and there is concern that the observed trend may not persist after treatment stopping.

The end of the blending interval informs the time beyond which there is no relative treatment effect. This can be approximated based on expert opinion or a targeted literature review of studies or appraisals on treatments with similar biological mechanisms. For example, literature reviews^8,11^ suggest that the NICE appraisal committee generally consider a 3- to 5-y treatment effect waning period to be plausible for IO treatments, which guided the choices in our case study.

Beta parameters have no direct physical interpretation, but it is important to understand their mathematical implications. Parameter a represents the degree of confidence in the internal model during the initial phase of the blending interval. When a is small, the blended hazard changes steeply right after $t_{1}$ . As a increases, the change becomes more gradual, and the blended hazard tends to follow the trend of the fitted internal hazard for a longer duration. Parameter b represents the degree of confidence in the external model during the final phase of the blending interval. When b is small, the blended hazard changes steeply right before $t_{2}$ . As b increases, the change becomes more gradual, and the blended hazard begins to converge to the fitted external hazard earlier. The trend of blended hazard relies more on the fitted internal hazard when a>b and more on the fitted external hazard when a<b .

**Figure 6:**
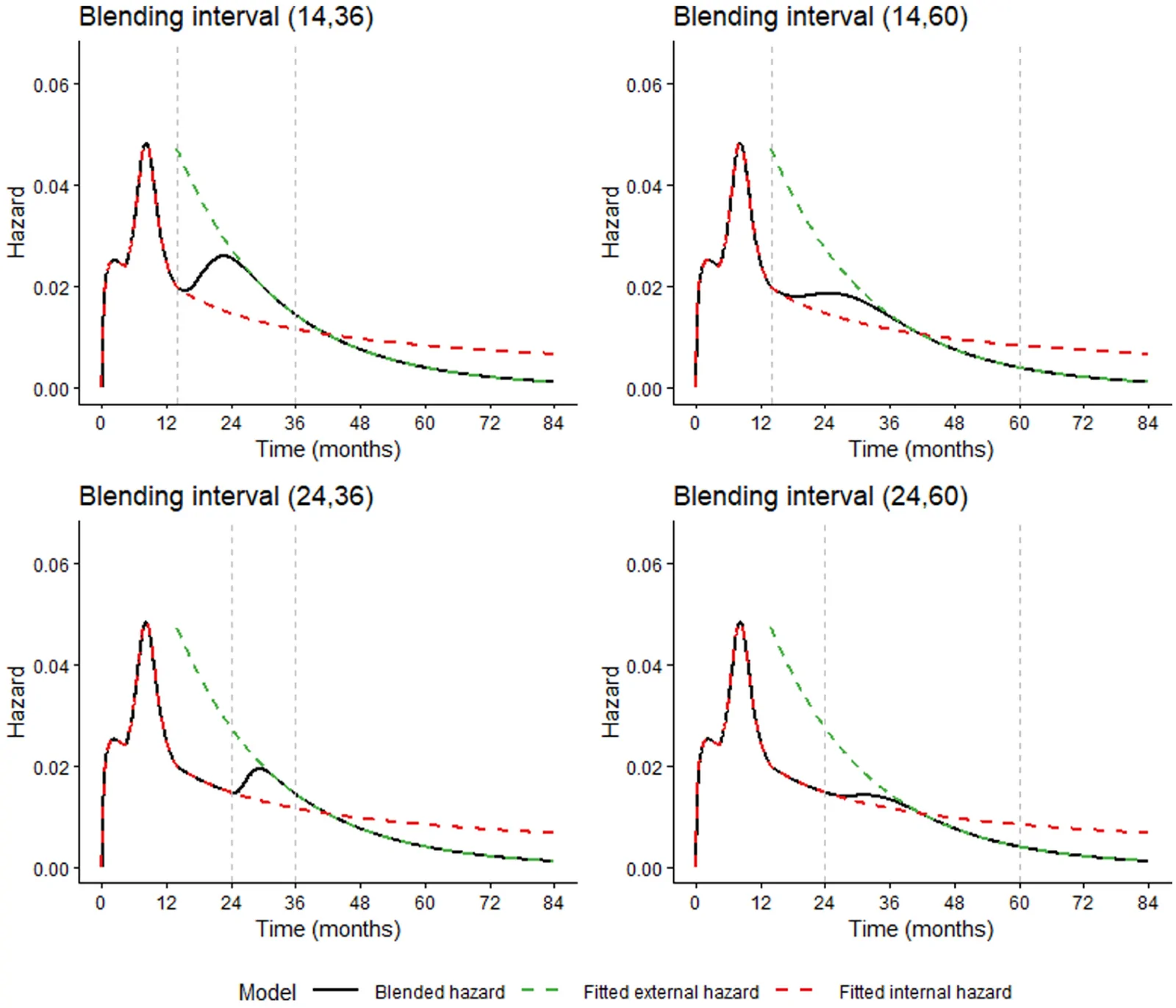
Blended hazard functions illustrating the sensitivity to different blending intervals. All scenarios use the same internal model, external model, and Beta parameters for this demonstration.

**Figure 7:**
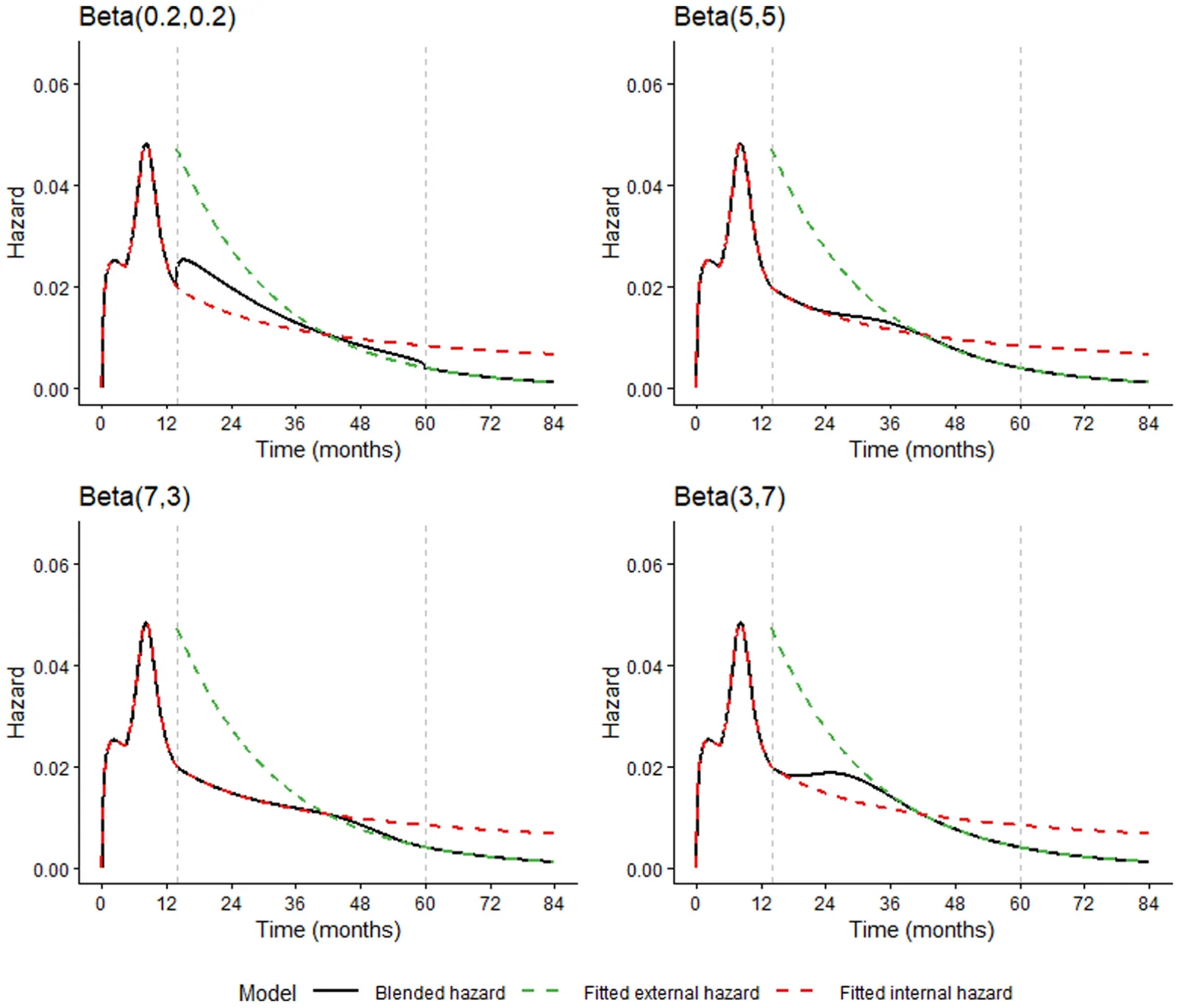
Blended hazard functions illustrating the sensitivity to different Beta parameters. All scenarios use the same internal model, external model, and blending interval for this demonstration.

Given the substantial uncertainty in treatment effect waning, we strongly advise modelers to explore a broad range of plausible blending intervals and Beta parameters within sensitivity analyses. The range of blending intervals can be determined as suggested above. However, we would caution against restricting Beta parameters a priori. The Beta parameters control how the blended hazard transitions from the fitted internal hazard to the fitted external hazard within the blending interval and therefore are specific to fitted models and not typically informed directly by clinical evidence. After implementing all sensitivity analysis scenarios, visual inspection of the blended hazard can be used as a post hoc check to assess their plausibility and exclude those producing clinically implausible projections. For example, Figure 6 presents blended hazard functions with different blending intervals and the same Beta parameters, and Figure 7 presents blended hazard functions with different Beta parameters and the same blending interval. In scenarios with short blending intervals or small Beta parameters, a drastic change in the blended hazard may be observed. Modelers should evaluate whether such changes are clinically plausible during the treatment-waning period. If so, they should also consider the appropriate magnitude of such changes. If the resulting survival function is sensitive to the choice of blending interval and Beta parameters, the most clinically plausible scenario can be selected as the base case, with alternative scenarios reported in sensitivity analyses. If not, all scenarios may be presented as plausible scenarios.

## Conclusions

There has been a noticeable gap between the growing demand for modeling treatment effect waning and the underdeveloped methods that appropriately address this issue. We demonstrate the blended hazard method as a possible approach to account for treatment effect waning while incorporating external evidence. The blended hazard method possesses the flexibility to accommodate a wide range of waning scenarios, thereby relaxing unnecessarily strong assumptions and effectively characterizing associated uncertainty in survival extrapolation. The application of this method in the NICE TA366 case study demonstrates consistent results with the updated 7-y follow-up data and shows significant improvement over the original TA366 base case using the same data at the time of appraisal in all scenarios. The results from this case study demonstrate that the type of waning assumptions commonly used in HTA may be too conservative in some cases and may actually underestimate the true treatment benefit. On the basis of this article, we encourage further validation of this method and call for more methods to be developed for modeling treatment effect waning in survival extrapolation.

## Supplemental Material

sj-pdf-1-mdm-10.1177_0272989X261452264 – Supplemental material for Flexible Survival Extrapolation with Blended Hazards: Accounting for Treatment Effect Waning in Health Technology Assessment Supplemental material, sj-pdf-1-mdm-10.1177_0272989X261452264 for Flexible Survival Extrapolation with Blended Hazards: Accounting for Treatment Effect Waning in Health Technology Assessment by Jingqi Zhu, Matthew Hemstock, Zhaojing Che, Gianluca Baio and Richard Birnie in Medical Decision Making
