# Supplementary material for: Flexible Survival Extrapolation with Blended Hazards: Accounting for Treatment Effect Waning in Health Technology Assessment
Source: Med Decis Making. 2026 Jun 19;46(7):848–62. doi: 10.1177/0272989X261452264 (PMC13601686; doi:10.1177/0272989X261452264)
Supplement: Supplementary file 1 [file sj-pdf-1-mdm-10.1177_0272989X261452264.pdf]

Supplementary materials

S1 Model selection

Model selection involves selection of models fitted to OS data from each arm of KEYNOTE-006 trial (internal data) and OS data from treatment-naive Schadendorf pooled analysis (external data). A flowchart summarising the entire model selection process is shown in Figure 1.

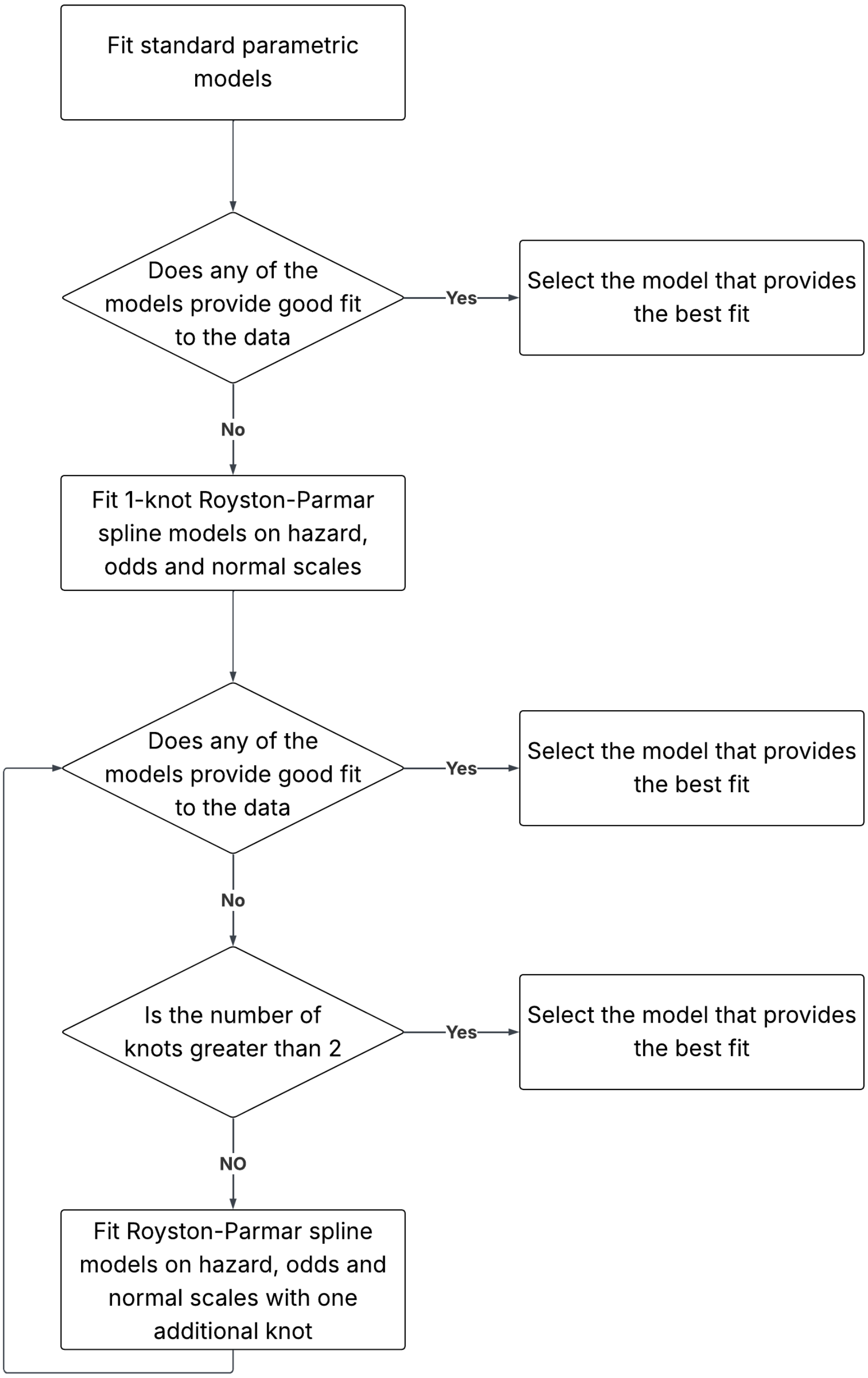

Figure 1 Flowchart of model selection process.

S1.1 Internal model selection

Pembrolizumab arm: 3-knot spline normal model

In the pembrolizumab arm, the nonparametric smoothed hazard derived from the data exhibited an initial increase followed by a decrease (Figure 2). Accordingly, generalised gamma, log-logistic, and log-normal models were first evaluated. However, none adequately reproduced the observed decline in smoothed hazard prior to the median follow-up, where the shape of smoothed hazard is generally more robust due to a larger number of patients at risk (Figure 3). The survival curves did not align well with the Kaplan–Meier estimate (Figure 4).

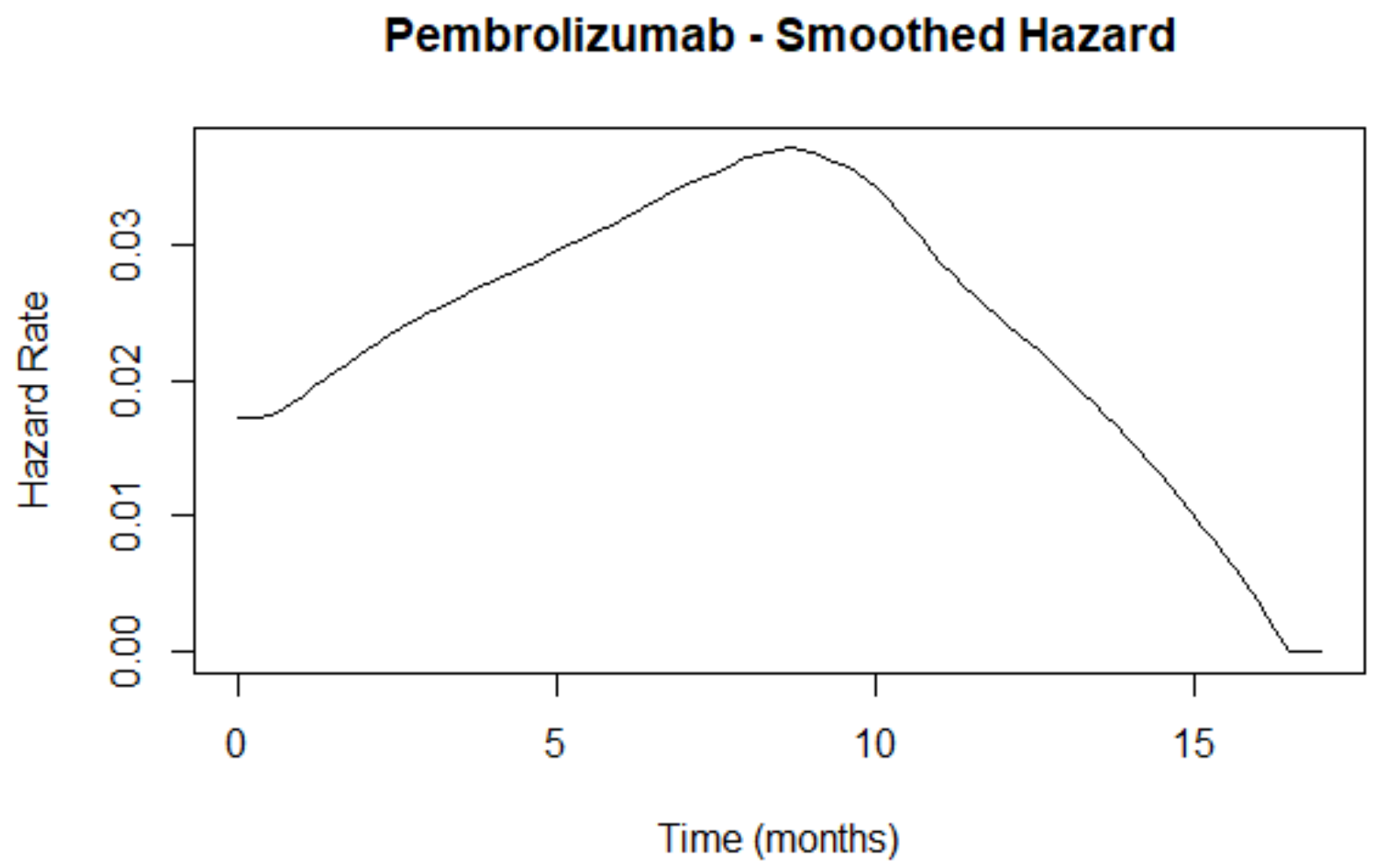

Figure 2 Nonparameteric smoothed hazard of the pembrolizumab arm presented an increasing-then-decreasing shape.

To allow greater flexibility, three Royston–Parmar spline models — on the hazard, odds and normal scales — with one knot were evaluated. The three 1-knot spline models produced hazard and survival estimates that differed only slightly from the standard parametric models, still indicating a lack of fit (Figure 5, 6).

The 2-knot spline models were more effective at capturing the decreasing trend, while the smoothed hazards closely approached the margins of 95% confidence intervals and these models appeared to overestimate the hazard from the 12th month to the median follow-up (Figure 7). The survival estimates still showed a lack of fit compared to the Kaplan–Meier estimate (Figure 8). As slight overfitting to observed trial data can be tolerated under the blended framework, a further escalation of model complexity was considered.

The 3-knot spline models better captured the general trend of hazard compared to the 2-knot spline models up to the median follow-up, though the wiggly hazard estimates suggested a slight overfitting (Figure 9). Survival curves from 3-knot spline models presented better consistency with the Kaplan–Meier estimate compared to the 2-knot spline models (Figure 10). The 3-knot spline models yielded significantly lower AIC values than the 2-knot spline models, whereas the differences in BIC were less pronounced (Table 1). We therefore considered the 3-knot spline models superior, and no need for further escalation of model complexity. The 3-knot spline normal model was ultimately selected for the pembrolizumab

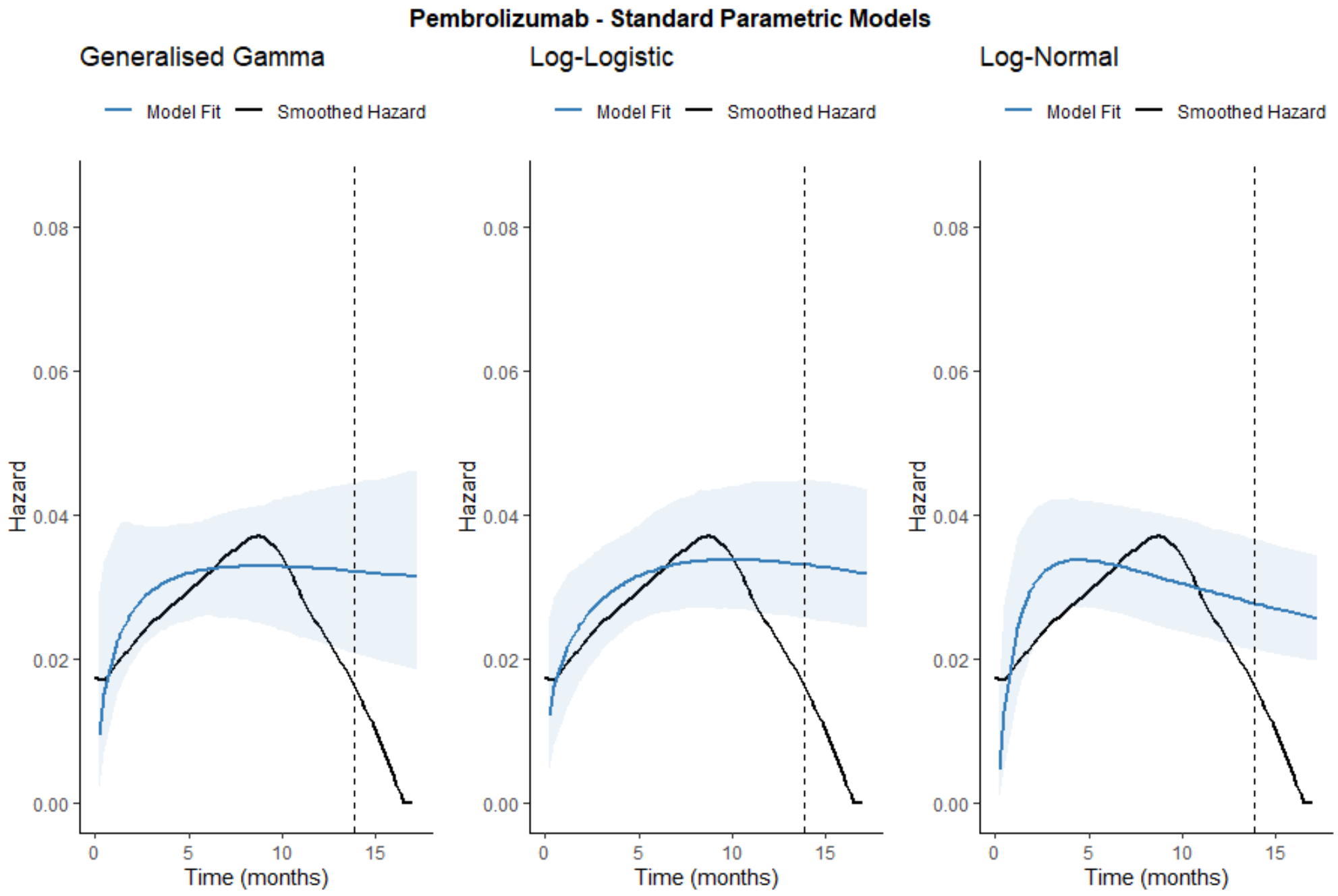

Figure 3 Generalised gamma, log-logistic and log-normal models could not reproduce the observed decline in smoothed hazard of the pembrolizumab arm prior to the median follow-up (the dashed line).

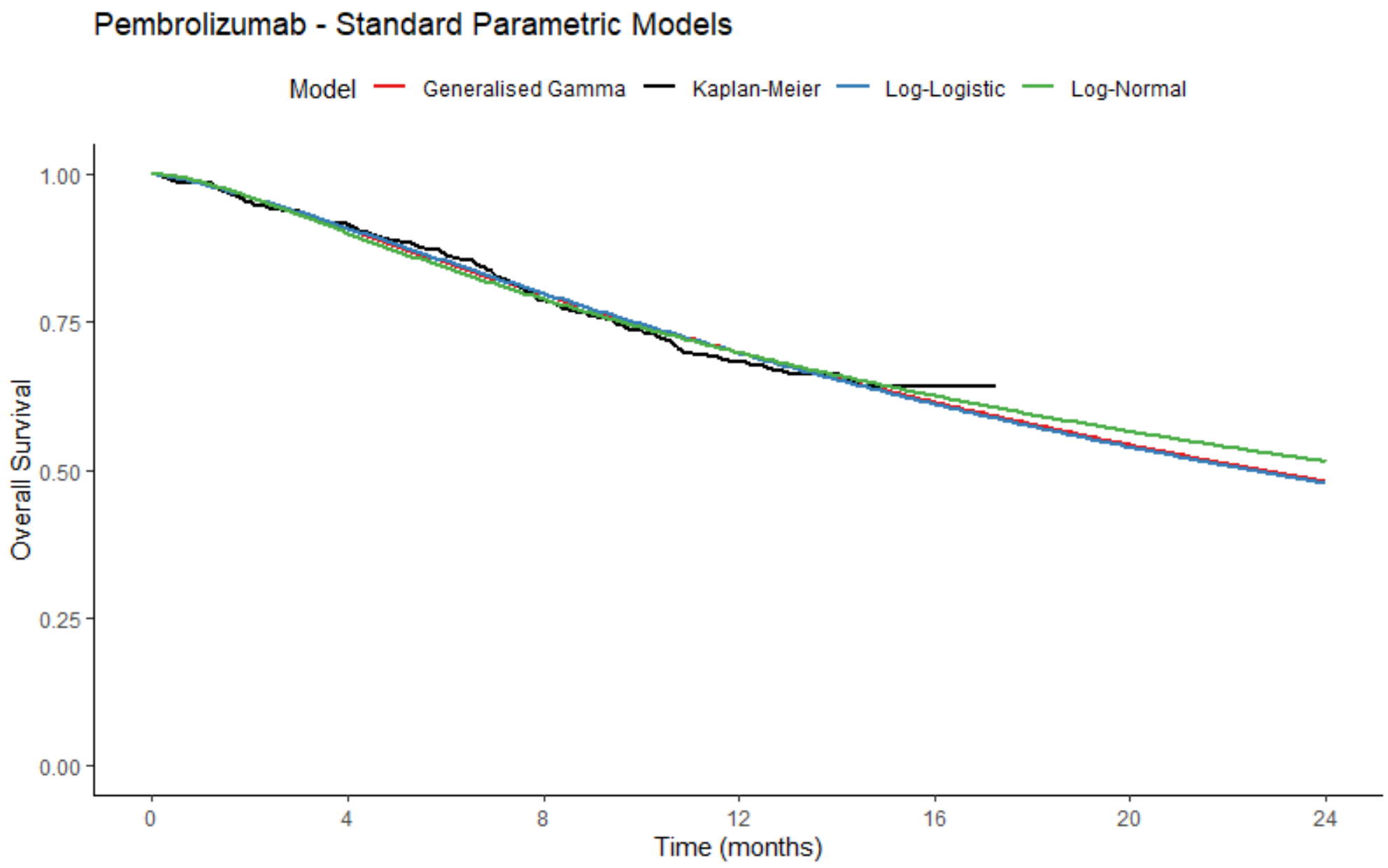

Figure 4 Survival curves from generalised gamma, log-logistic and log-normal models did not align well with the Kaplan–Meier estimate of the pembrolizumab arm.

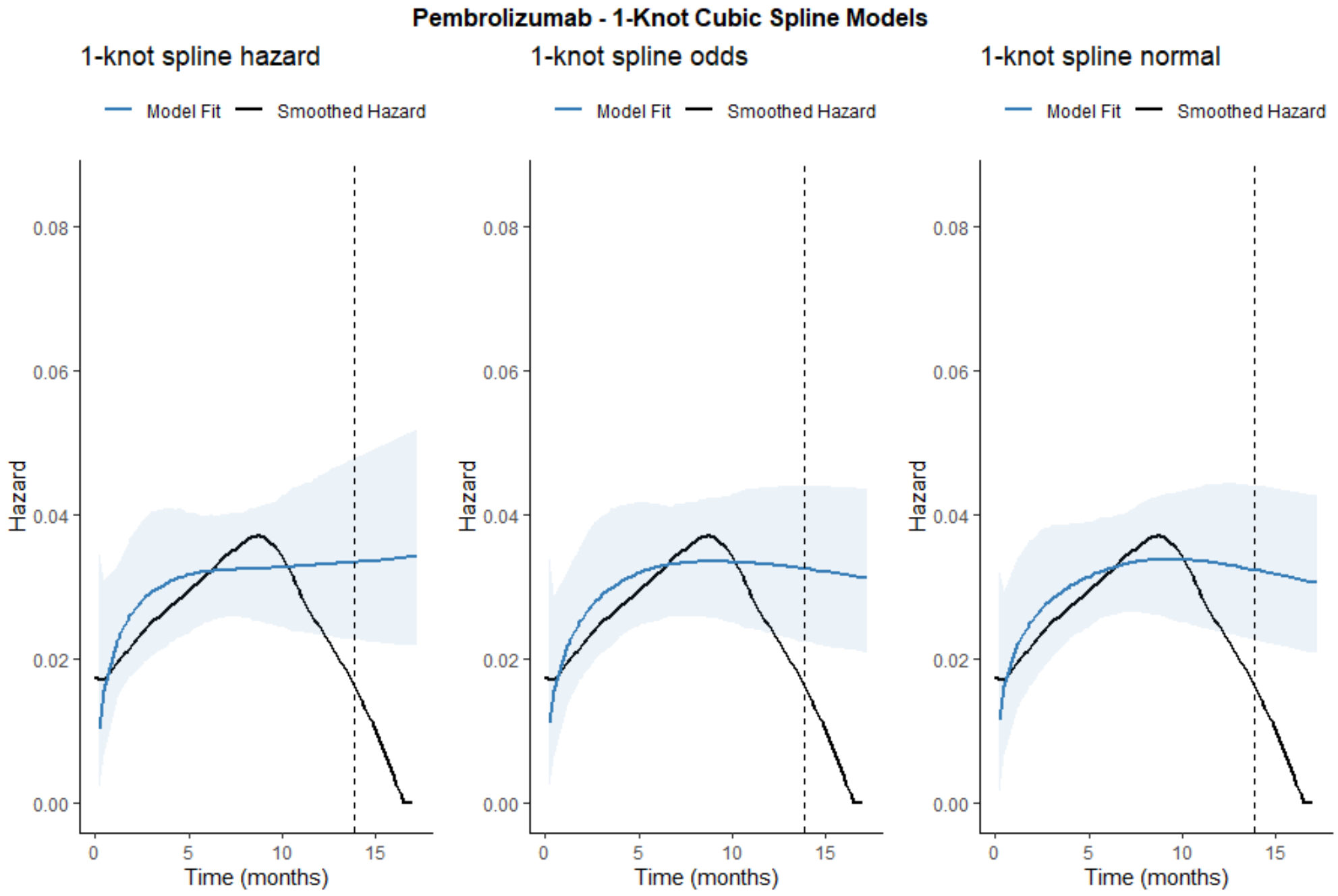

Figure 5 Hazard estimates from 1-knot spline models could not reproduce the observed decline in smoothed hazard of the pembrolizumab arm prior to the median follow-up (the dashed line).

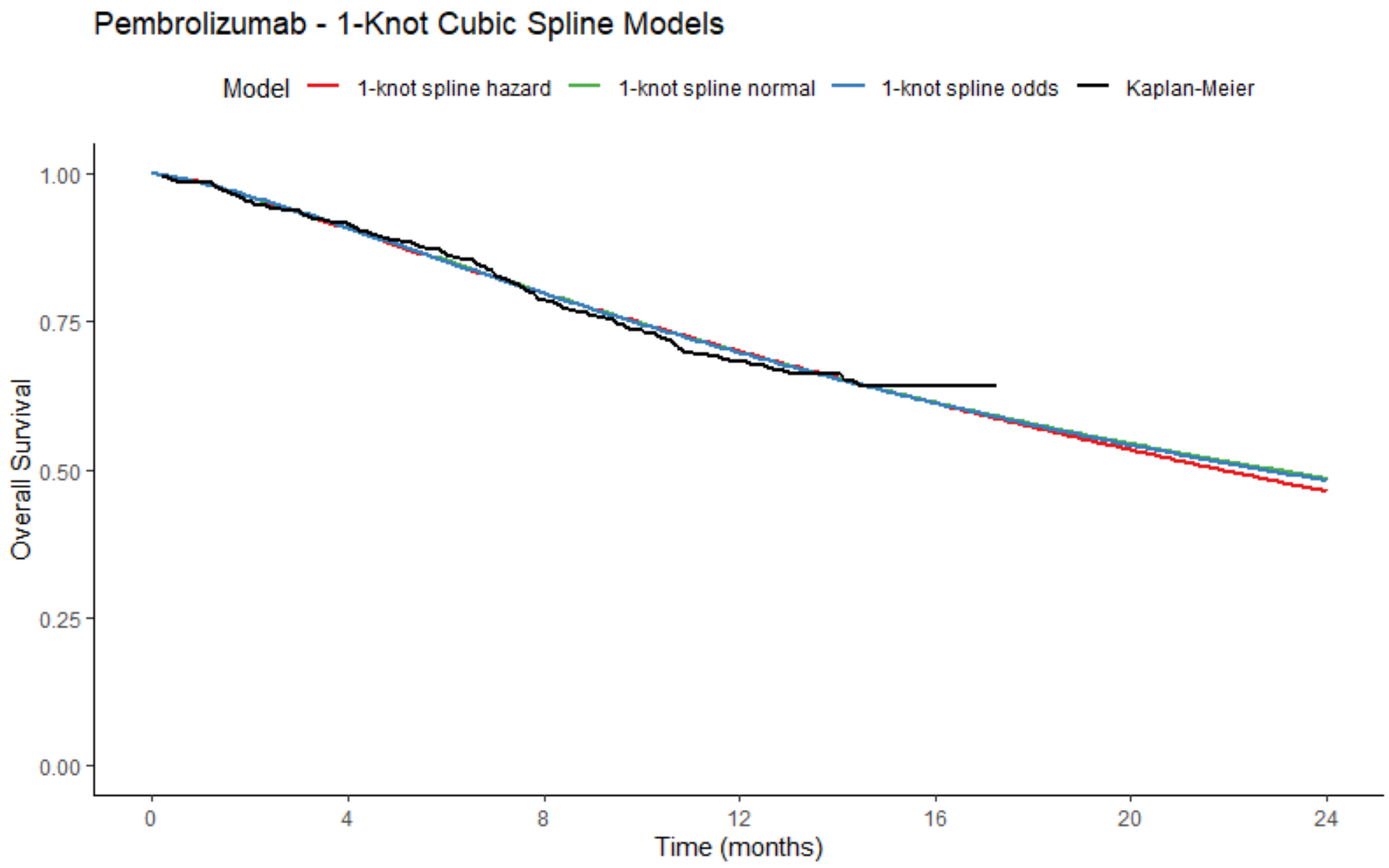

Figure 6 Survival curves from 1-knot spline models did not align well with the Kaplan–Meier estimate of the pembrolizumab arm.

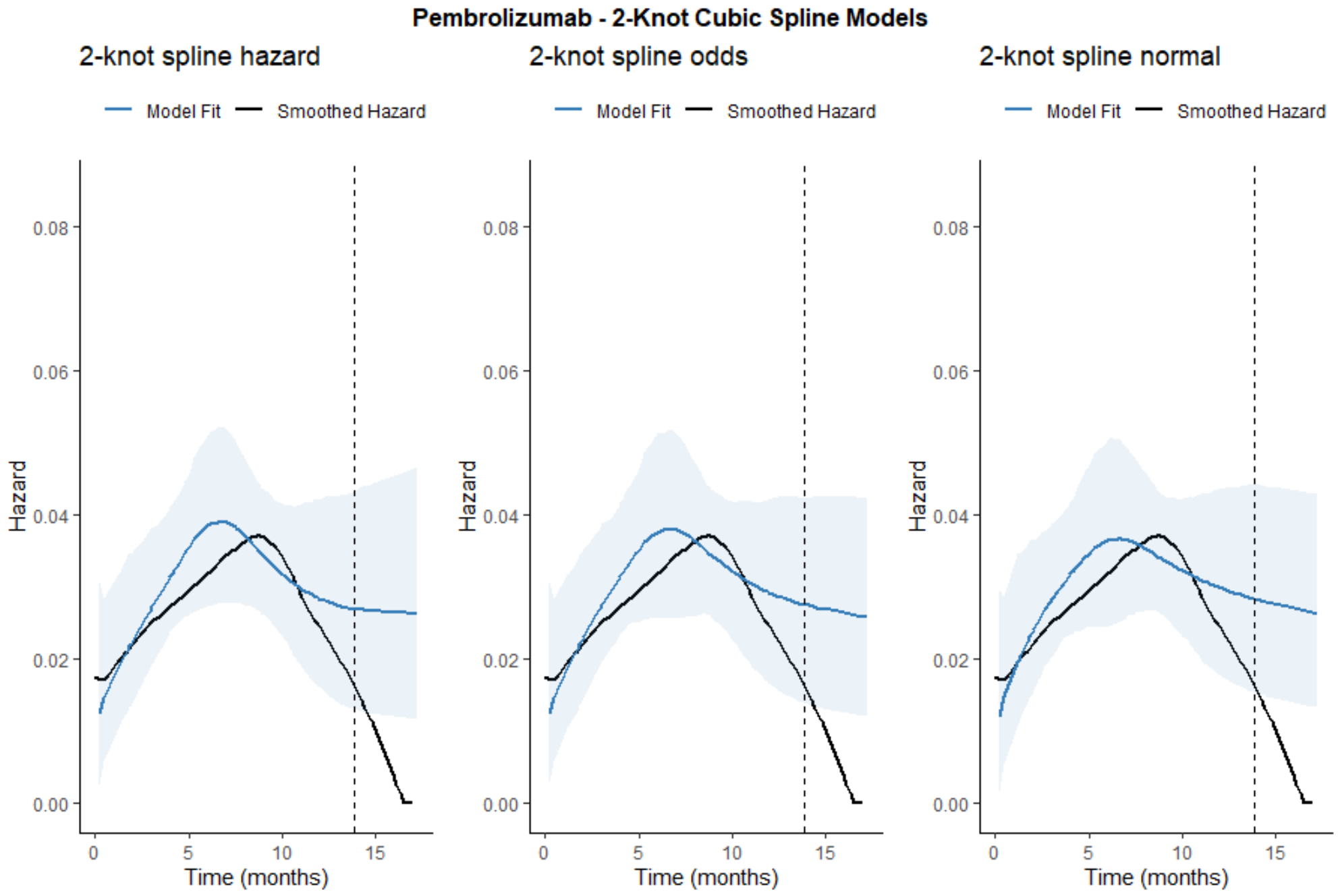

Figure 7 Hazard estimates from 2-knot spline models captured the decreasing trend in smoothed hazard of the pembrolizumab arm, but still overestimating hazard from the 12th month to the median follow-up (the dashed line).

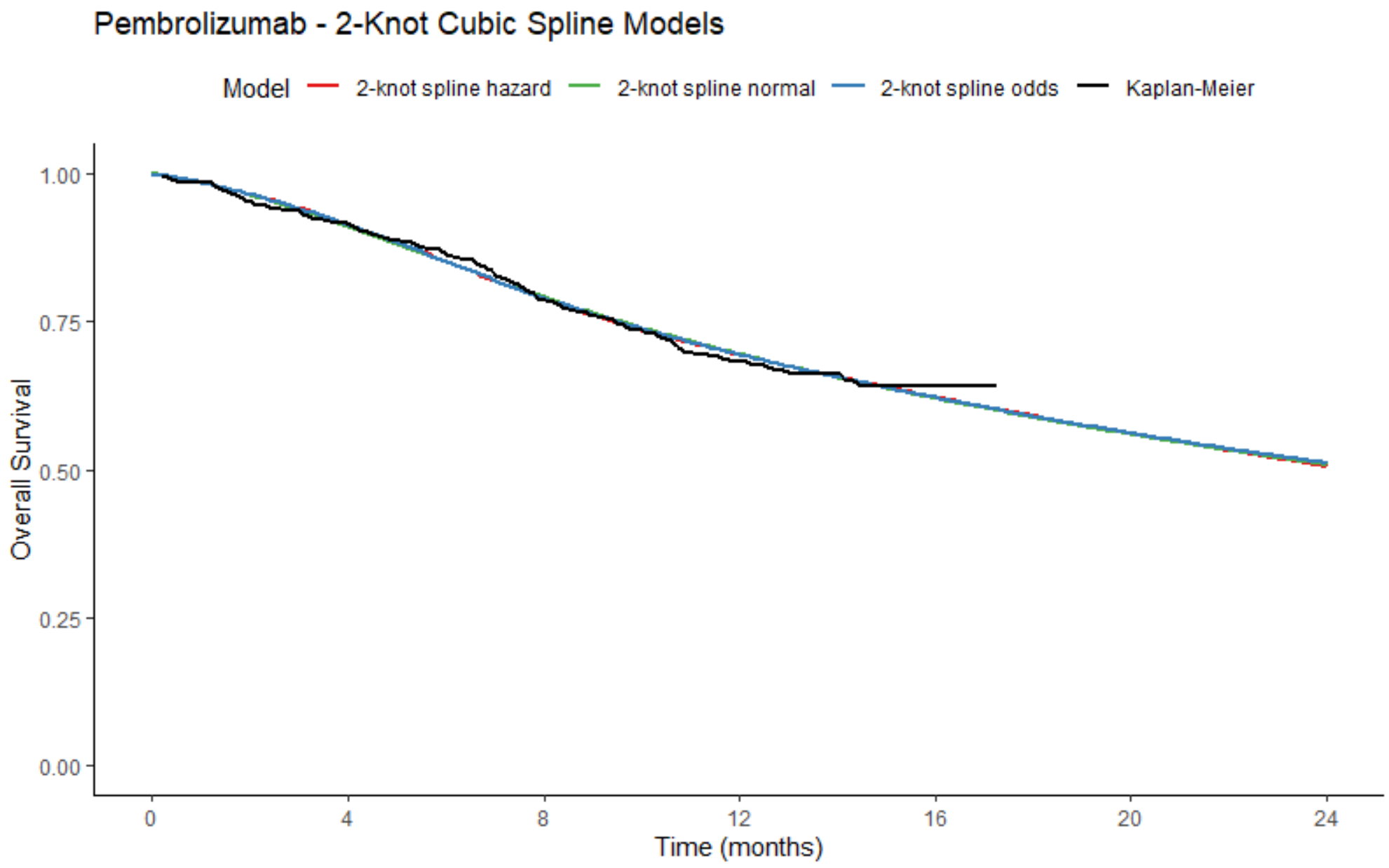

Figure 8 Survival curves from 2-knot spline models still demonstrated a slight lack of fit compared to the Kaplan–Meier estimate of the pembrolizumab arm.

arm, as it achieved the most favourable AIC and BIC values among the three 3-knot spline models (Table 1).

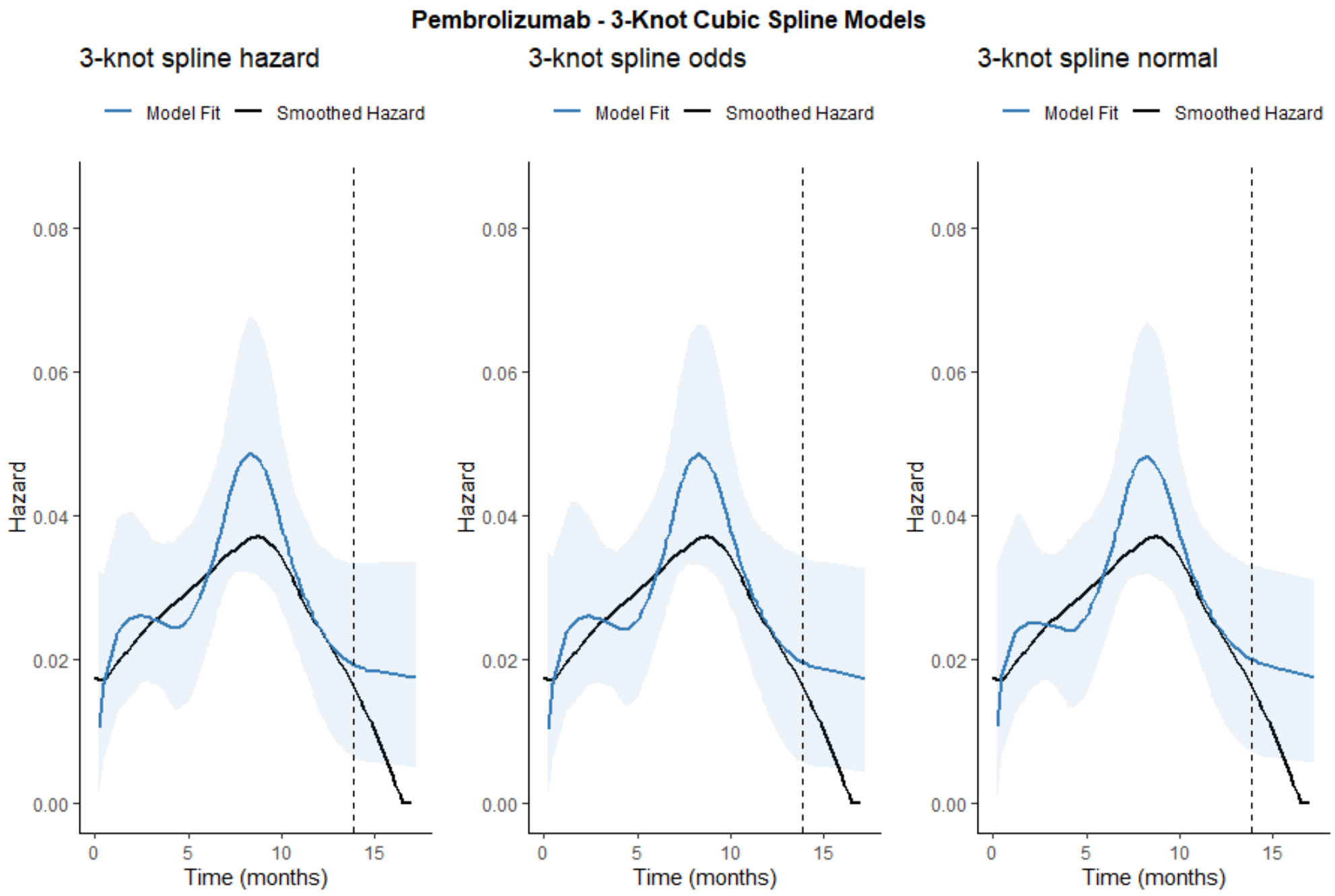

Figure 9 The 3-knot spline models better captured the general trend of hazard of the pembrolizumab arm compared to the 2-knot spline models up to the median follow-up (the dashed line), though the wiggly hazard estimates suggested a slight overfitting.

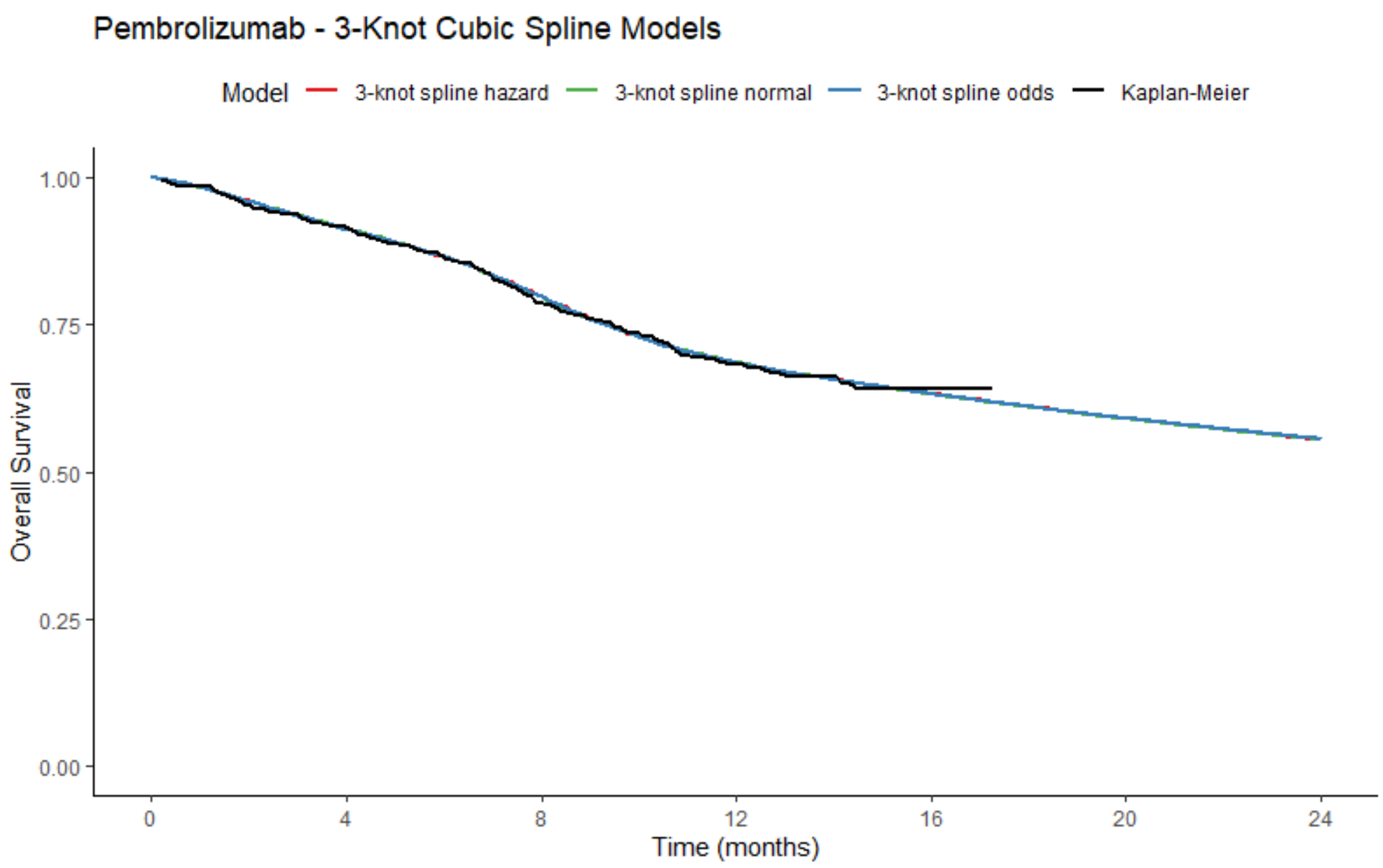

Figure 10 Survival curves from 3-knot spline models presented good consistency with the Kaplan–Meier estimate of the pembrolizumab arm.

Model selection for the ipilimumab arm followed a similar procedure. Given the increasing-then-decreasing shape of the smoothed hazard (Figure 11), log-

Table 1: AIC and BIC values of models evaluated for the internal model fit to the pembrolizumab arm data. The 3-knot spline models yielded significantly lower AIC values than the 2-knot spline models, whereas the differences in BIC were less pronounced. The 3-knot spline normal model achieved the lowest favourable AIC and BIC values among all 3-knot spline models.

Model	AIC	BIC
Generalised Gamma	833.10	843.98
Log-Logistic	830.89	838.14
Log-Normal	832.43	839.67
1-knot spline hazard	833.60	844.47
1-knot spline odds	832.87	843.74
1-knot spline normal	832.49	843.37
2-knot spline hazard	833.84	848.34
2-knot spline odds	833.89	848.39
2-knot spline normal	833.94	848.44
3-knot spline hazard	831.83	849.95
3-knot spline odds	831.78	849.90
3-knot spline normal	831.74	849.86

logistic, log-normal, and generalised gamma models were first evaluated. The log-logistic and log-normal models failed to capture the decline in hazard before the median follow-up, resulting in overestimation in survival relative to the Kaplan–Meier estimate (Figure 12). The generalised gamma model provided a generally reasonable fit on both hazard (Figure 12) and survival (Figure 13) scale. It also achieved lower AIC values than the log-logistic and log-normal models (Table 2).

Ipilimumab arm: generalised Gamma model

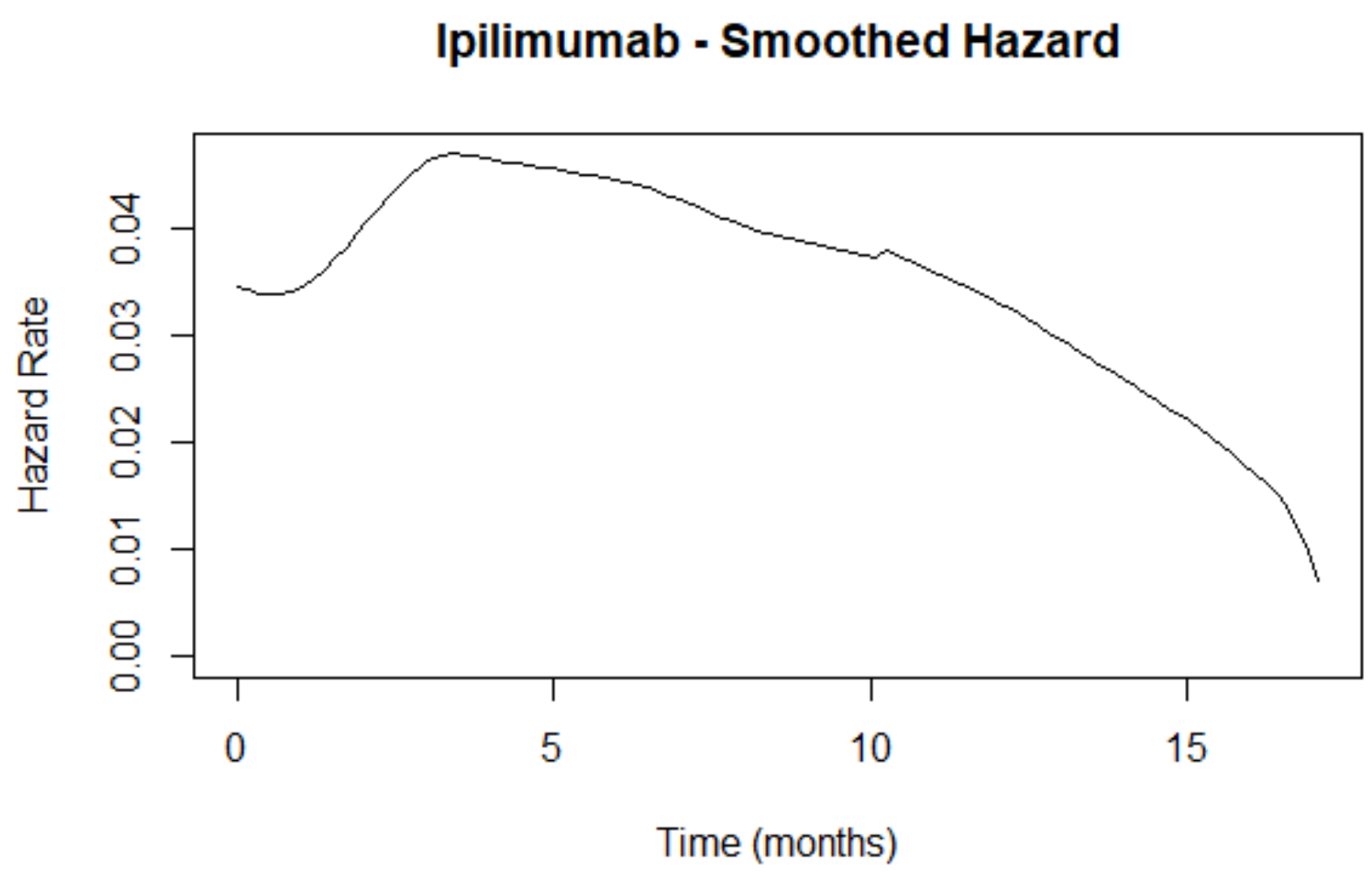

Figure 11 Nonparameteric smoothed hazard of the ipilimumab arm presented an increasing-then-decreasing shape.

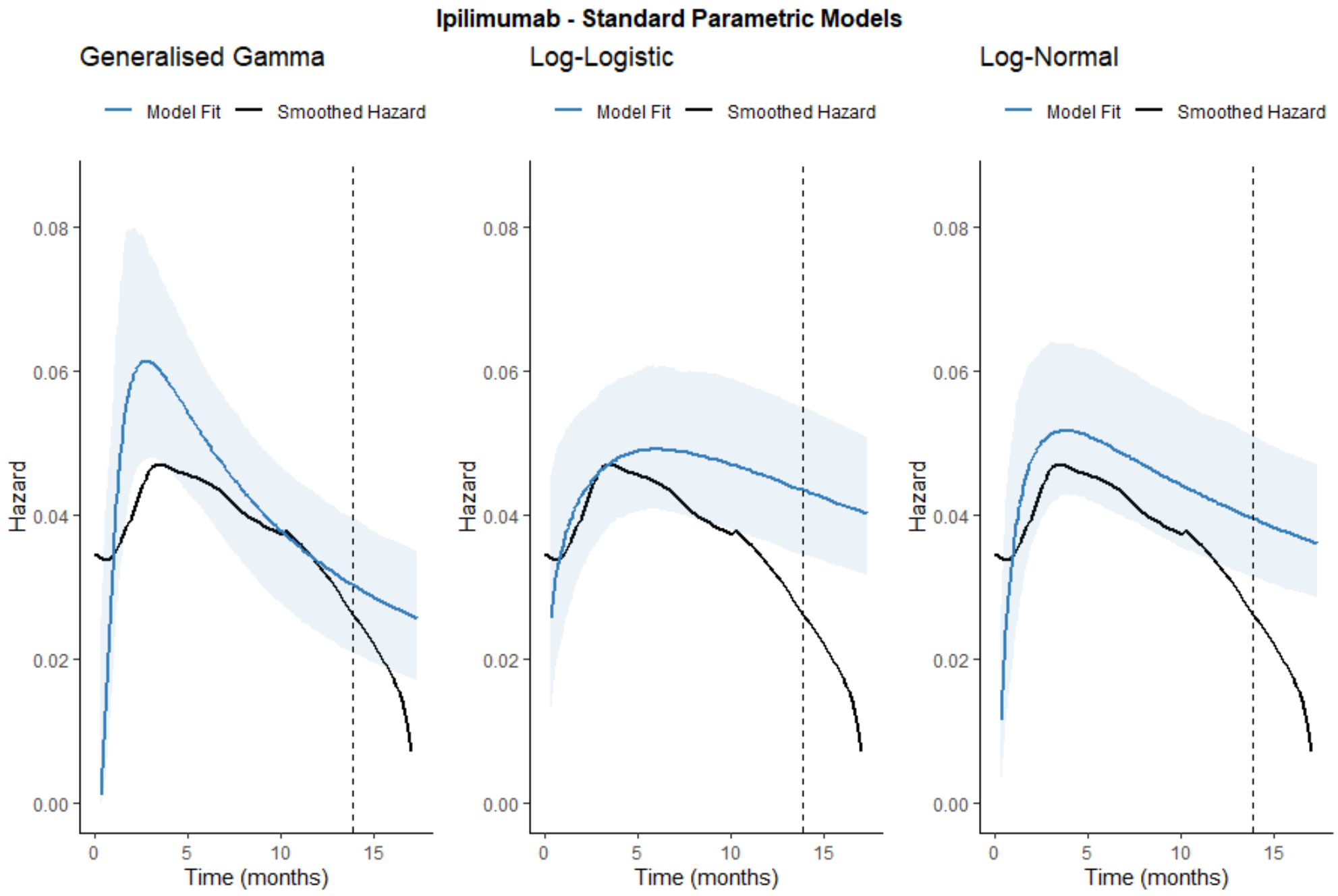

Figure 12 Log-logistic and log-normal models significantly overestimated hazard of the ipilimumab arm prior to the median follow-up (the dashed line). The generalised gamma model generally captured the trend of hazard.

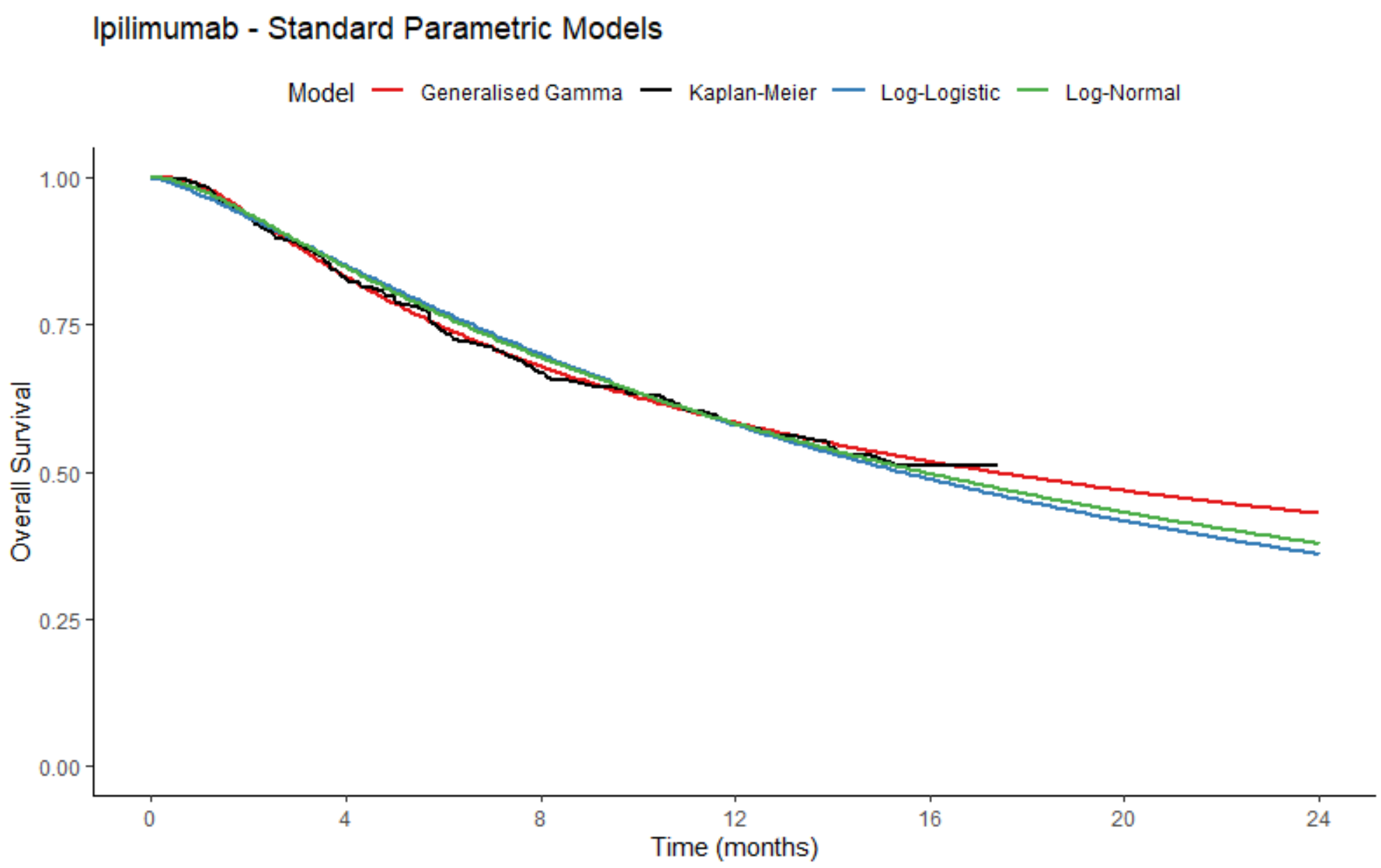

Figure 13 The survival curve from the generalised gamma model aligned closely with the Kaplan–Meier estimates for the ipilimumab arm, whereas the log-logistic and log-normal models showed poorer consistency.

To assess whether further escalation of model flexibility was need, we fitted 1-knot Royston–Parmar spline models on the hazard, odds, and normal scales. Although all three 1-knot spline models fitted the data well from visual inspection of hazard (Figure 14) and survival (Figure 15) plots, there was no substantial improvement over the generalised gamma model. Furthermore, the AIC and BIC values of the spline models were higher than the generalised gamma model (Table 2), indicating the escalation of model complexity was not worthwhile. Consequently, the generalised gamma model was selected for the ipilimumab arm.

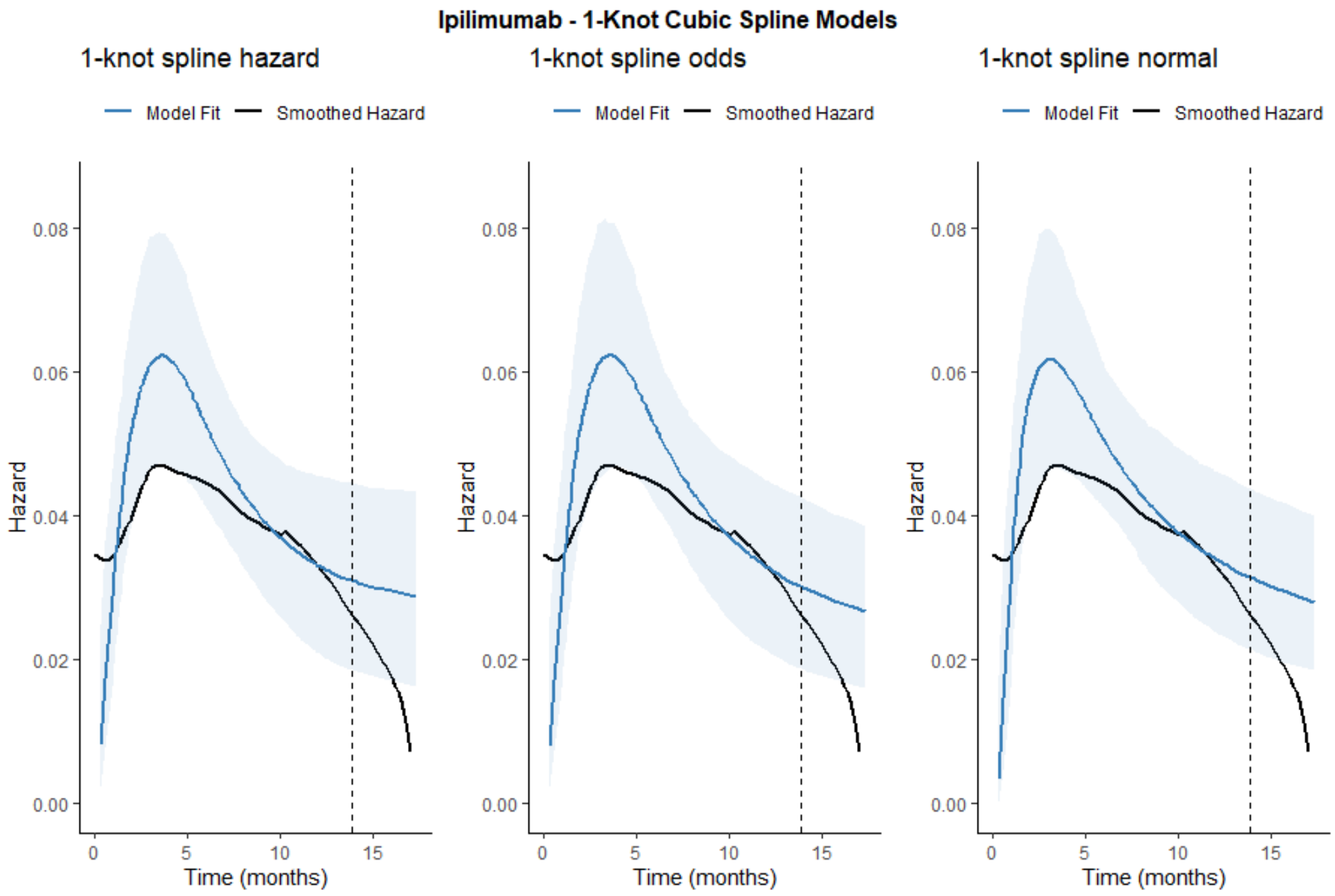

Figure 14 Hazard estimates from 1-knot spline models generally captured the trend in smoothed hazard of the ipilimumab arm prior to the median follow-up (the dashed line).

Table 2: AIC and BIC values of models evaluated for the internal model fit to the ipilimumab arm data. The generalised gamma model yielded the lowest AIC value and the second-lowest BIC value, with only a minor difference from the lowest BIC.

Model	AIC	BIC
Generalised Gamma	922.87	933.75
Log-Logistic	931.27	938.52
Log-Normal	924.79	932.05
1-knot spline hazard	925.00	935.88
1-knot spline odds	924.96	935.84
1-knot spline normal	923.52	934.40

S1.2 External model selection

The external model is intended to fit to the data from Schadendorf pooled analysis, particularly beyond the start of blending interval. We first explored standard

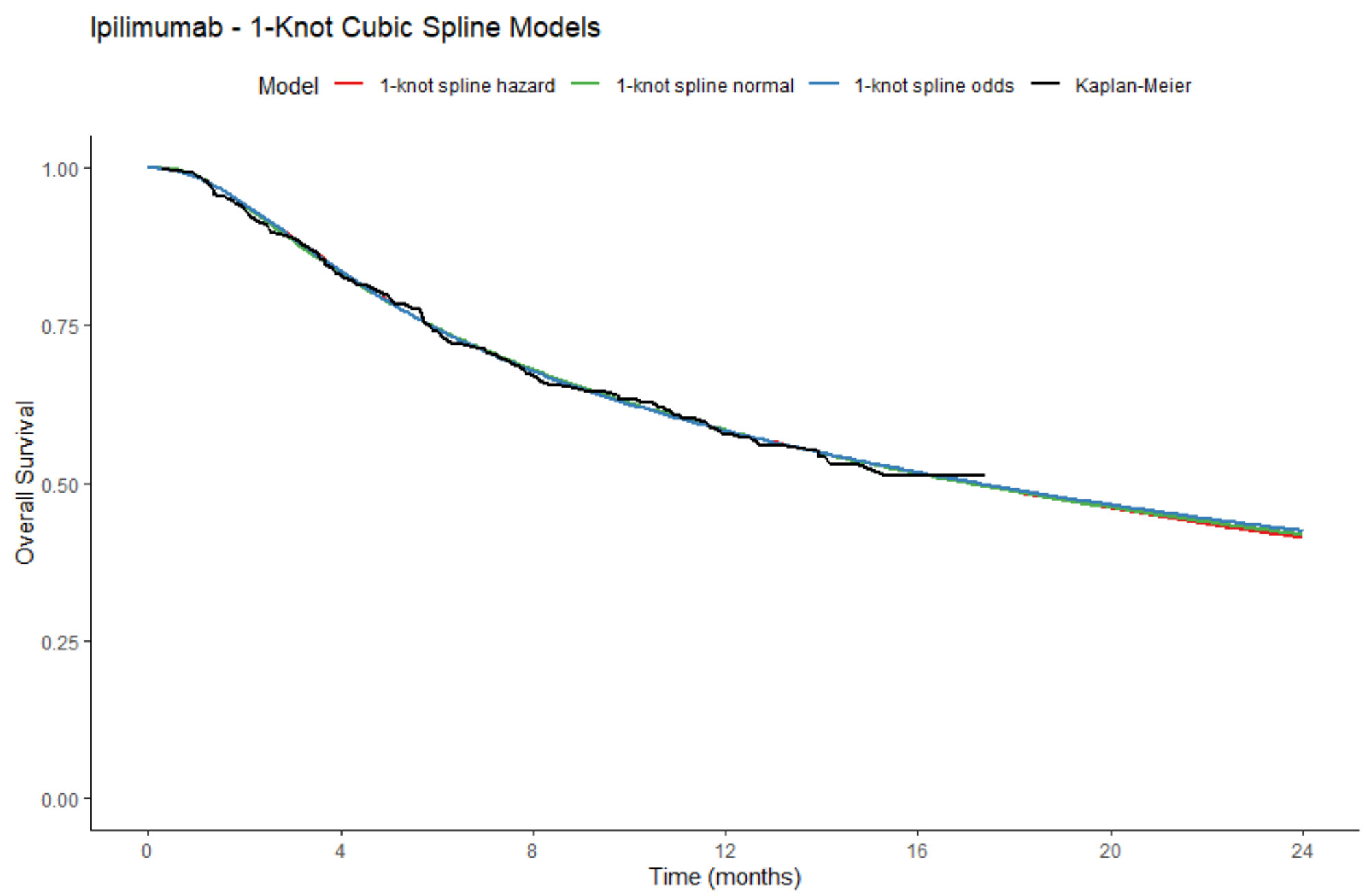

Figure 15 Survival curves from 1-knot spline models aligned well with the Kaplan–Meier estimate of the ipilimumab arm.

parametric models from randomisation. Given the general decreasing shape of the smoothed hazard (Figure 16), Weibull, Gompertz, log-logistic log-normal and generalised gamma distributions were evaluated and compared. By visual inspection of hazard and survival plots, the best model was a Gompertz model that underestimated hazard before 42 months and overestimated hazard from 42 to 60 months (17). This led to overestimation of survival before 56 months and underestimation thereafter (18). We believed that this model fit was not good enough in capturing mid-term and long-term hazard of the external data, and the bias due to lack of fit should not be propagated into the blending process.

Then, standard parametric models rebased at the median follow-up of the internal KEYNOTE-006 trial (13.84 months) were considered, as the Kaplan–Meier of the external data were supported by a substantial number of events beyond this time, and we believed the hazard estimate from the internal model should be reliable enough to not blend in any external information before this time point. The rebased Gompertz model well captured the decreasing trend in the smoothed hazard of the external data, while other models did not (Figure 19). The rebased Gompertz model also showed the best consistency with the Kaplan–Meier estimate of the external data (Figure 20) and yielded the lowest AIC and BIC values among all fitted models (Table 3). Thus, the Gompertz model rebased at 13.84 months was selected as the external model.

S2 Base case selection

We aimed to select the model representing the most clinically plausible hazard and survival projection as the base case (hazard and survival plots for 16 models can be accessed at `https://github.com/JZhu919/BlendTrtWaning/tree/main/figures`). None of the models generated implausible survival projections. Seven

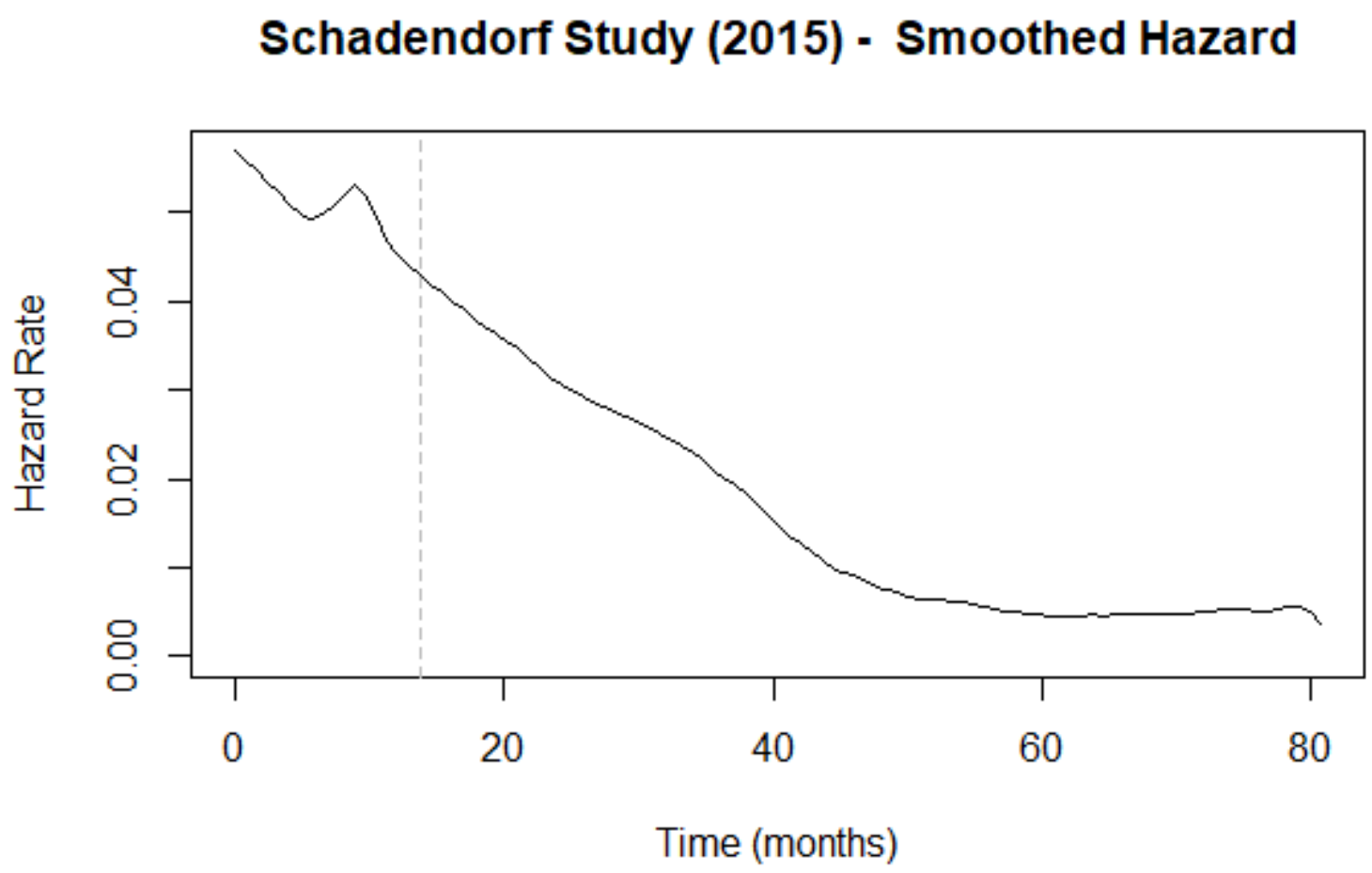

Figure 16 Nonparametric smoothed hazard of the external data has a decreasing shape beyond the median follow-up of the internal trial (the dashed line).

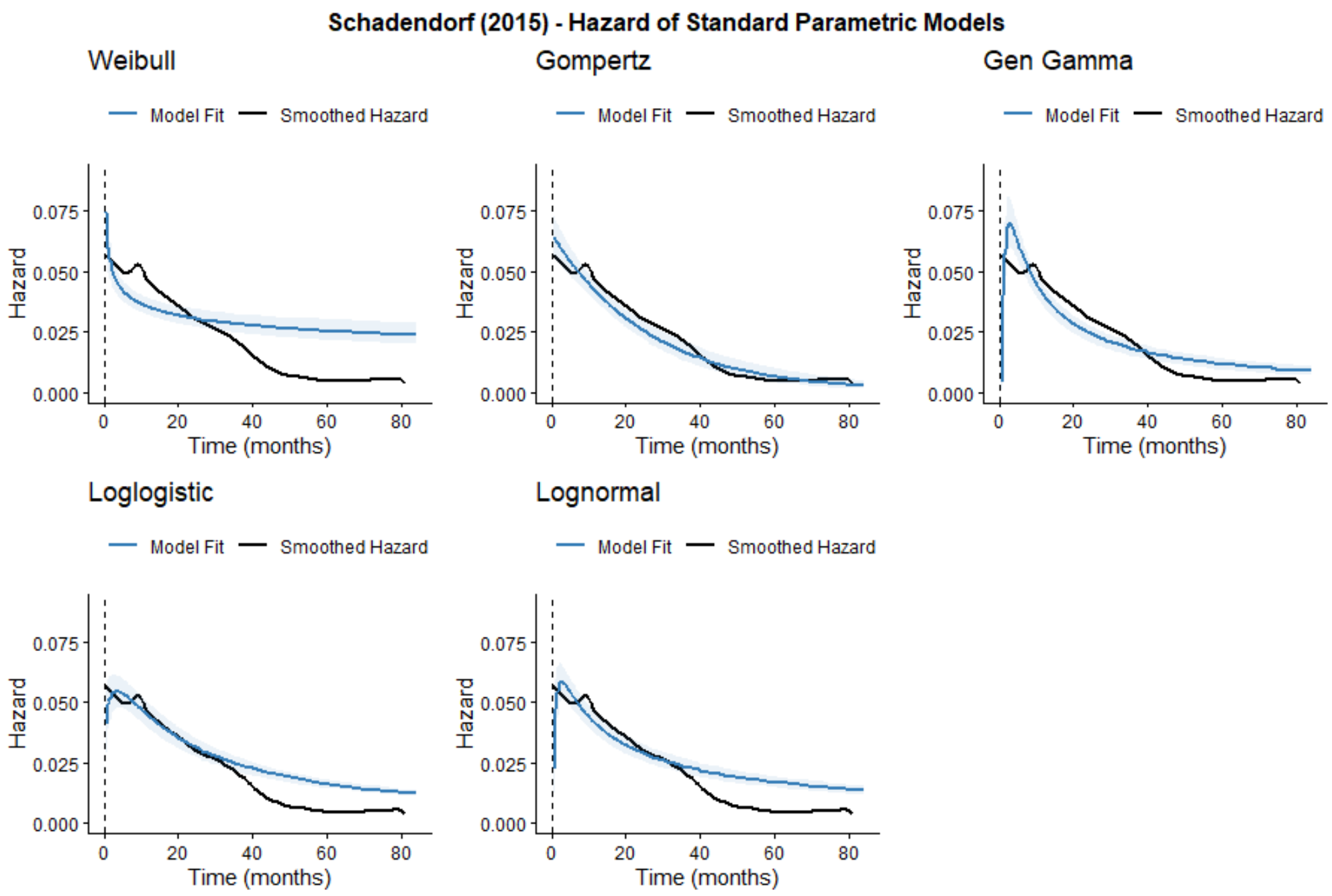

Figure 17 Hazard estimates from standard parametric models compared to the smoothed hazard of the external data. The Gompertz model generally captured the decreasing trend in the smoothed hazard of the external data, while underestimated hazard before 42 months and overestimated hazard from 42 to 60 months.

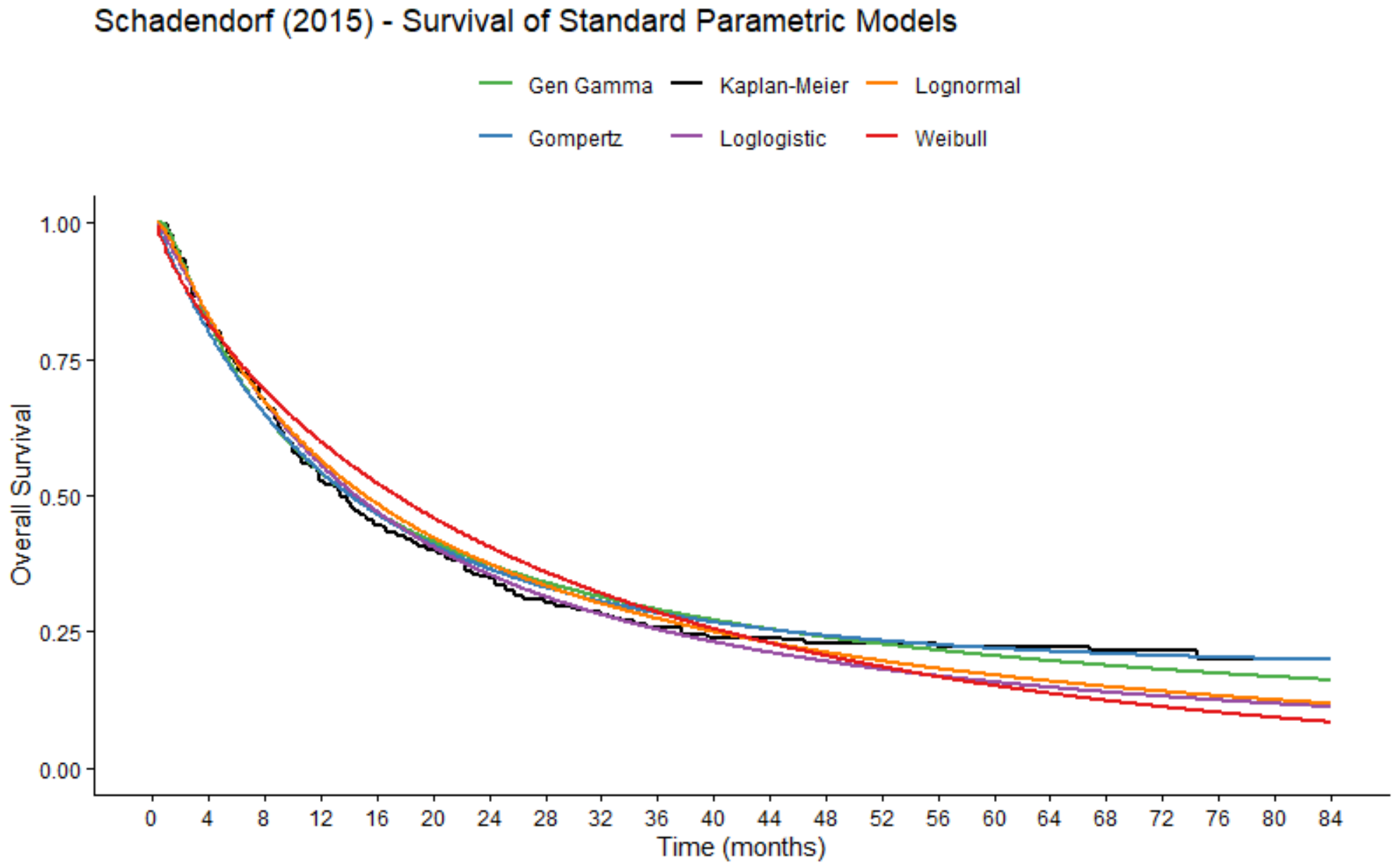

Figure 18 Survival curves from standard parametric models compared to the Kaplan–Meier estimate of the external data. None of the models showed good enough consistency with the Kaplan–Meier estimate.

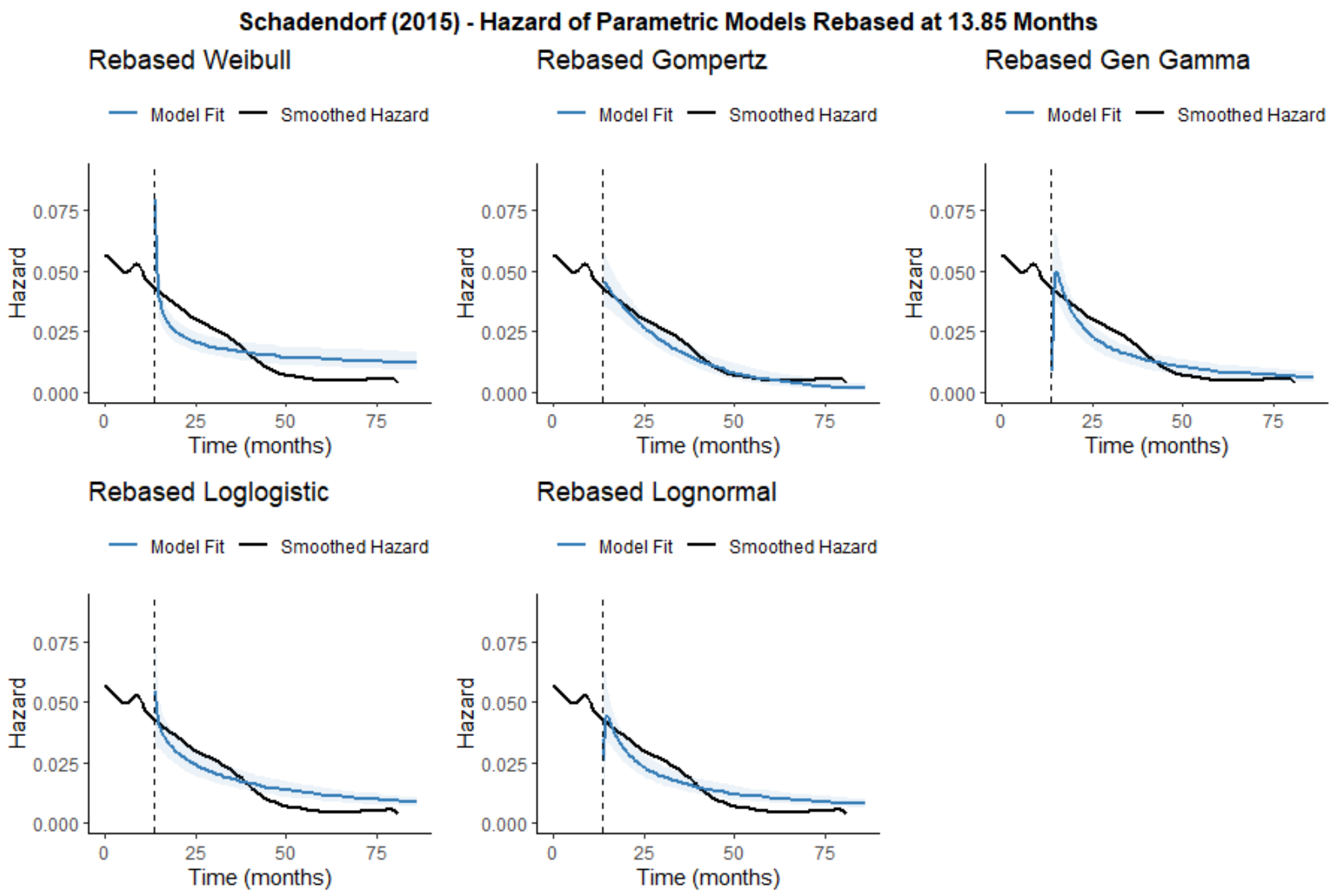

Figure 19 Hazard estimates from standard parametric models rebased at the median follow-up of the internal trial (the dashed line) compared to the smoothed hazard of the external data. The rebased Gompertz model well captured the decreasing trend in the smoothed hazard of the external data, while other models did not.

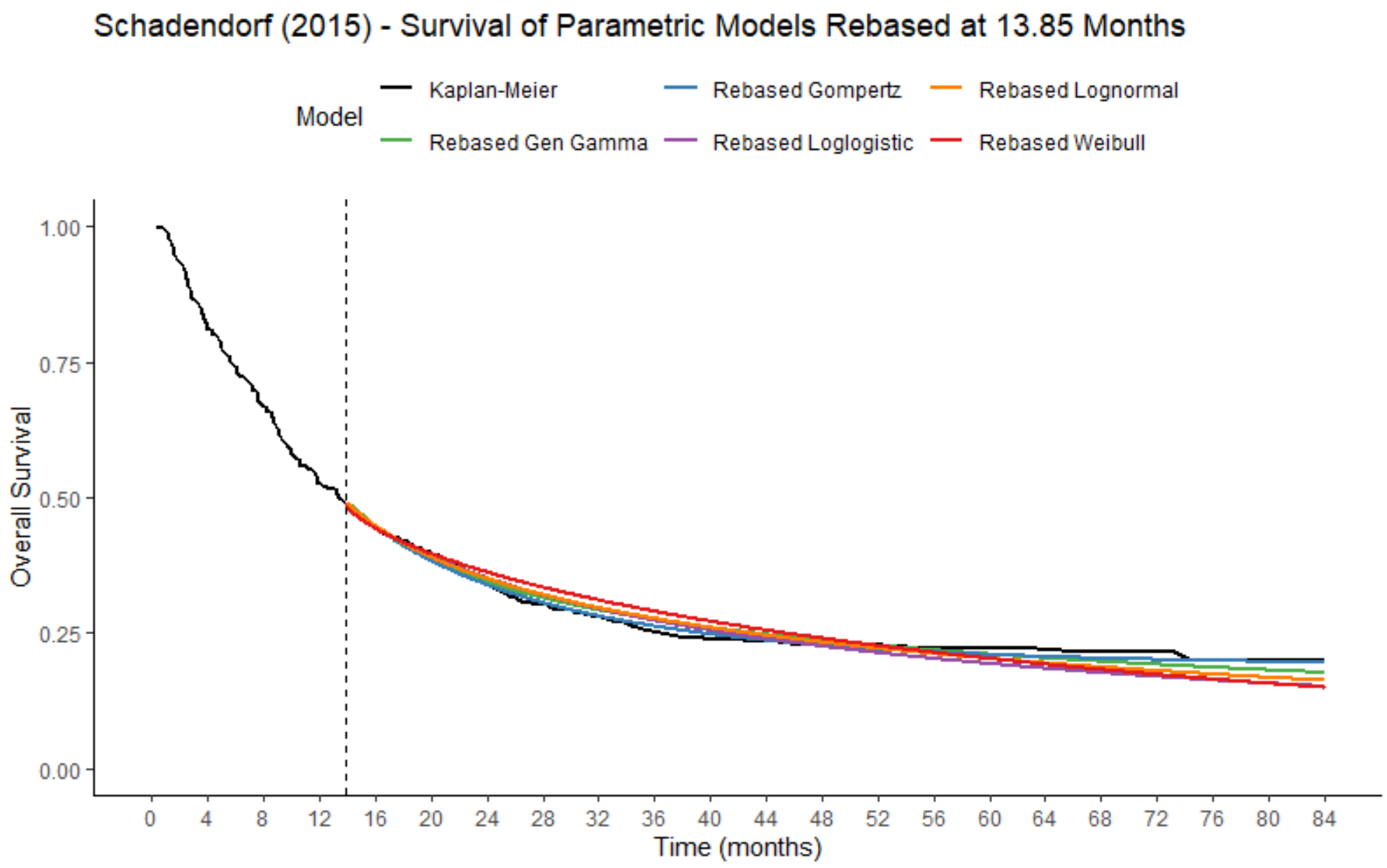

Figure 20 Survival curves from standard parametric models rebased at the median follow-up of the internal trial (the dashed line) compared to the Kaplan–Meier estimate of the external data. The rebased Gompertz model showed the best consistency with the Kaplan–Meier estimate.

Table 3: AIC and BIC values of models evaluated for the internal model fit to the external data. The rebased Gompertz model yielded the lowest AIC and BIC values among all rebased models. Note, AIC/BIC of rebased and non-rebased models are not directly comparable as they are not calculated based on the same data.

Model	AIC	BIC
Weibull	3355.97	3364.76
Gompertz	3281.25	3290.05
Generalised Gamma	3290.73	3303.92
Log-Logistic	3297.92	3306.71
Log-Normal	3293.49	3302.28
Rebased Weibull	984.32	991.49
Rebased Gompertz	971.93	979.10
Rebased Generalised Gamma	980.46	991.21
Rebased Log-Logistic	977.89	985.05
Rebased Log-Normal	978.77	985.94

models with narrow blending intervals (24–36 months) or very small Beta parameters (a = 0.2, b = 0.2) were first ruled out for base case consideration, because they produced substantial fluctuations in the hazard around the start and the end of the blending interval. Given the clinical context, we considered such pronounced fluctuations less likely to reflect true underlying long-term hazard. The remaining nine models produced either no or more clinically reasonable fluctuations in hazard within the blending interval. Informed by the fact that there was a pre-defined 2-year stopping rule, and that equalising hazards five years after the start of treatment is the most used assumption in past NICE IO appraisals, the base case was selected as the model with blending interval $(24, 60)$ months and Beta parameters $(5, 5)$. i.e. weight function $\pi(t \mid t_1 = 24, t_2 = 60, a = 5, b = 5)$. The remaining 15 models were included as sensitivity analyses.
